# Bis(7)-harmine derivatives as potential multi-target anti-Alzheimer agents

**DOI:** 10.3389/fchem.2025.1545908

**Published:** 2025-01-29

**Authors:** Hongtao Du, Fang Ma, Yuanyuan Cao, Miaoyan Bai, Xinyi Gao, Ziyi Yang, Yang Xu, Yan Yan

**Affiliations:** ^1^ Shaanxi Key Laboratory of Chinese Jujube, College of Life Sciences, Yan’an University, Yan’an, Shaanxi, China; ^2^ Shaanxi Qi Yuan Kang Bo Biotechnology Co., Ltd., Tongchuan, Shaanxi, China; ^3^ College of Life Science, Xinyang Normal University, Xinyang, China; ^4^ Northwest A&F University, Xianyang, Shaanxi, China

**Keywords:** Alzheimer’s disease, harmine, acetylcholinesterase, monoamine oxidase, amyloid peptide (Aβ)

## Abstract

**Introduction:**

The multi-targeted ligands (MTDL) strategy has been recognized as a promising Approach for the development of effective treatments against Alzheimer’s disease (AD), due to the presence of multiple pathological mechanisms in AD. In this study, a series of bis(7)-harmine derivatives were designed and synthesized as multifunctional drugs for the treatment of AD.

**Methods:**

The derivatives were synthesized by chemical methods and their structure was confirmed by nuclear magnetic resonance (NMR). The Ellman’s assay was utilized to assess the inhibitory potential of derivatives against *h*AChE and *h*BuChE. The inhibitory activity of these derivatives on both *h*MAO-A and *h*MAO-B was assessed using a fluorescence-based method. The thioflavin T (Th-T) fluorescence assay was used to assess the inhibition of A*β*_1−42_ self-aggregation. The cytotoxicity was evaluated using the MTT assay. The Surflex-Dock program in Sybyl-X2.0 Software was employed for molecular docking.

**Results:**

*In vitro* studies revealed that numerous synthesized compounds exhibited potent inhibitory activity against *h*AChE, and *h*MAO-B (IC_50_ < 1 μM), as well as A*β*_1−42_ aggregation (IC_50_ < 20 μM). Importantly, the multitarget compounds **6d**, **8c**, and **8d** exhibited remarkable efficacy in simultaneously mitigating A*β*-induced toxicity in SH−SY5Y cells while demonstrating minimal cytotoxicity. Furthermore, predicted ADMET results suggested that **6d**, **8c**, and **8d** possessed favorable pharmacokinetic properties and demonstrated low toxicity levels. Additionally, molecular docking studies of 6d within the activesites of *h*AChE, *h*MAO-B, and A*β*_1−42_ elucidated the inhibition mechanism.

**Discussion and conclusion:**

Based on these findings, it is evident that **6d**, **8c**, and **8d** hold potential as promising multi-functional drugs for AD treatment.

## 1 Introduction

Alzheimer’s disease (AD) is a progressive and irreversible neurodegenerative disorder that leads to a significant number of fatalities. Clinically, the primary cognitive symptoms of AD include progressive short-term memory loss, disorientation, and neuropsychological disturbances (Mendiola-Precoma et al., 2016). According to the World Health Organization (WHO) report, the global prevalence of AD is alarming, currently affecting nearly 45 million individuals, with projections indicating that this figure could surpass 150 million by 2050 (Prince et al., 2016). To date, the US Food and Drug Administration (FDA) has approved only four small molecule drugs (rivastigmine, galantamine, donepezil, and memantine) and two immunotherapy drugs (aducanumab and lecanemab) for the treatment of AD. However, these four drugs offer only symptomatic relief, while the two immunotherapy drugs are limited to treating mild-to-moderate AD and are associated with significant adverse effects, raising concerns about their safety and efficacy (Tan et al., 2014; Terao and Kodama, 2024). The current state of AD treatment is indeed a cause for serious concern, highlighting the urgent need for intensified efforts in the research and development of more effective therapies. In addition to the three main hypotheses—the cholinergic, amyloid-*β* (A*β*), and tau protein hypotheses—considered the primary pathological pathways of AD, several other targets have emerged, such as neuroinflammation and oxidative stress, many of which are interconnected (Hsu et al., 2023; Nasb et al., 2024). Given the complex and multifactorial nature of AD pathogenesis, current drug discovery research is shifting towards multitarget strategies aimed at simultaneously affecting multiple nodes of the intricate neurodegenerative network.

In the amyloid cascade hypothesis, the accumulation of A*β* peptides is regarded as a crucial factor in the onset and progression of Alzheimer’s disease. The neurotoxic effects of A*β* are diverse and multifaceted. For example, A*β* oligomers can bind to neuronal surface receptors, disrupting synaptic function and plasticity, which are essential for learning and memory processes (Jeremic et al., 2021). Moreover, A*β* can induce oxidative stress and the generation of reactive oxygen species (ROS), leading to further neuronal damage. Over time, these toxic effects accumulate, resulting in substantial impairments in cognitive functions, including memory, attention, and executive function (Kepp et al., 2023; Zhang et al., 2023). Due to the pivotal role of A*β* in the pathogenesis of AD, significant research efforts have been devoted to developing effective strategies to either reduce A*β* production or enhance its clearance from the brain (Yadollahikhales and Rojas, 2023).

Acetylcholine (ACh), a major neurotransmitter in the central nervous system (CNS), plays a crucial role in the neurological regulation of various functions. In AD, ACh depletion is associated with cognitive deficits, arousing the cholinergic hypothesis in the physiopathology of AD and thus the search for inhibitors of its degrading enzymes, acetylcholinesterase (AChE) and butyrylcholinesterase (BuChE). Both AChE and BuChE are present in the brain with different specificities and expression activities. A selective inhibition of AChE is more crucial in the early stage, while BuChE inhibition may be critical in the mid to later stages of the pathogenesis (Abeysinghe et al., 2020). Selective inhibition of AChE is particularly important in the early stages of AD, whereas inhibition of BuChE becomes more critical in the mid to late stages of the disease (Marucci et al., 2021). Beyond its catalytic function, AChE also influences non-cholinergic processes through its peripheral anionic site (PAS) and contributes to the aggregation and progression of amyloid proteins (Ahmed et al., 2021; Marucci et al., 2021; Tonelli et al., 2023). Consequently, AChE remains a primary therapeutic target.

Monoamine oxidases (MAOs) constitute a family of mitochondrial enzymes that selectively catalyze the oxidative deamination of various biogenic and xenobiotic amines, including neurotransmitters such as 5-HT, dopamine, norepinephrine, and epinephrine (Cai, 2014; Riederer and Youdim, 1986). The significance of MAOs in neurobiology and pharmacology is highlighted by their role in modulating the levels of these neurotransmitters, thus affecting mood, behavior, and other physiological functions (Behl et al., 2021). Two distinct isoforms of monoamine oxidase exist: MAO-A and MAO-B. These isoforms exhibit differences in substrate specificity and inhibitor sensitivity. Selective MAO-A inhibitors have been widely acknowledged for their effectiveness in treating mood disorders, such as depression and anxiety. In contrast, MAO-B inhibitors are predominantly used in the management of neurological conditions to mitigate the production of neurotoxic substances, thereby safeguarding neuronal integrity and promoting neuronal survival (Banerjee et al., 2024). Importantly, activated MAO-B exacerbates neurodegenerative processes by elevating H_2_O_2_ levels, leading to oxidative stress, and by modulating A*β* production through *γ*-secretase activity in neurons (Schedin-Weiss et al., 2017). Consequently, a compound with dual inhibitory actions on AChE, MAO-B, and A*β* aggregation holds promise as a therapeutic agent for Alzheimer’s disease (AD).

**FIGURE 1:**
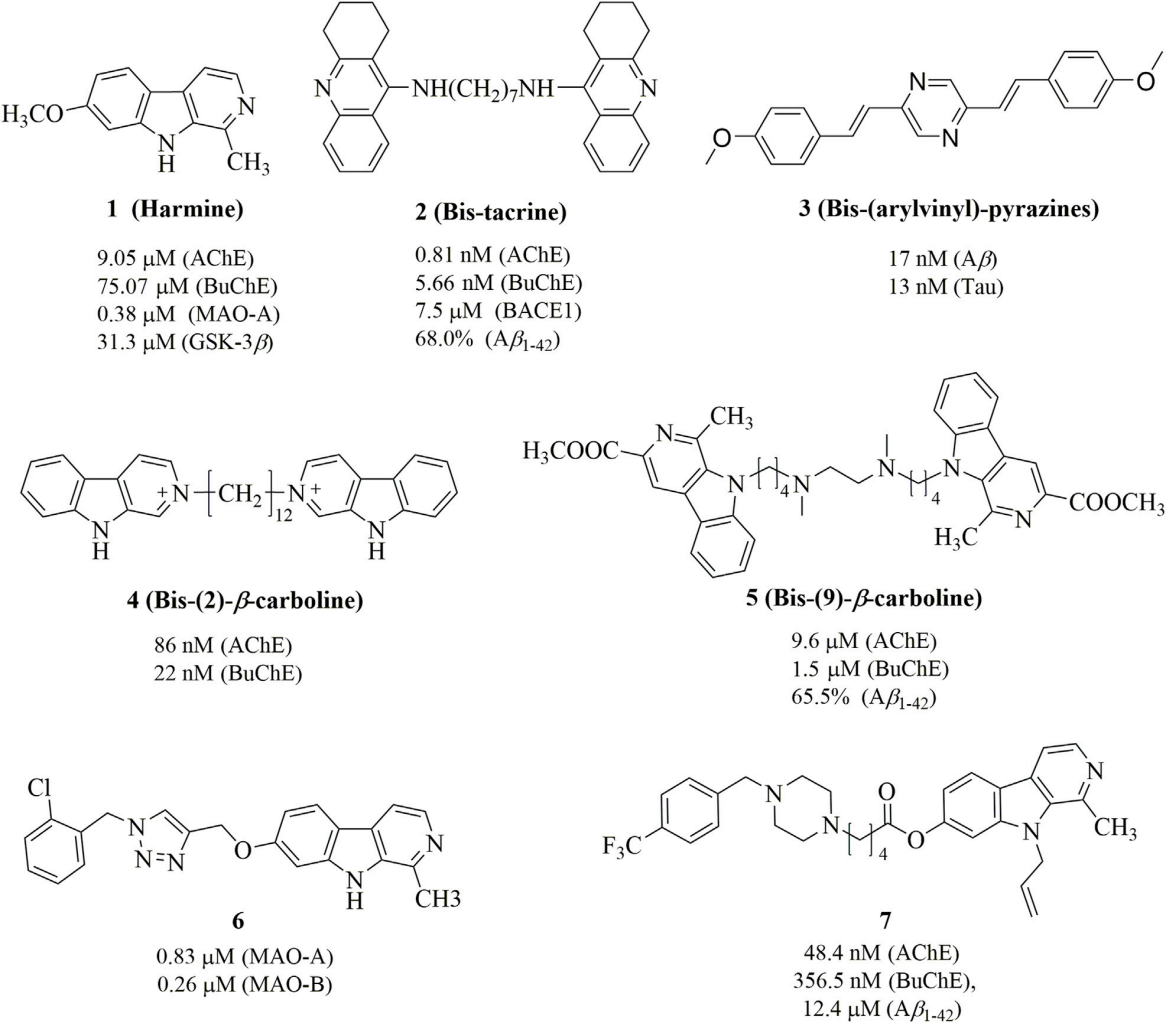
Chemical structures of harmine and its derivatives.

Harmine (Figure 1), a naturally occurring *β*-carboline alkaloid, exhibits a broad spectrum of biological activities, including antimicrobial, antitumor, antiviral, and antiparasitic properties (Patel et al., 2012; Zhang et al., 2020). Its core structure consists of a tricyclic pyrido [3,4-*b*]indole ring, which resembles human tryptamine-based neurotransmitters such as serotonin and melatonin. This structural similarity, combined with the increased rigidity provided by the additional ring, has been leveraged in the design of bioactive compounds to modulate various CNS targets (Warren et al., 2024). In particular, harmine and its derivatives have been reported as inhibitors of several biomolecular targets implicated in AD, such as A*β* aggregation, AChE, MAOs, 5-hydroxytryptamine (5-HT) and N-methyl-D-aspartate (NMDA) (Beato et al., 2021; Li et al., 2023). Therefore, with the emergence of multitarget strategies against AD, the harmine scaffold is naturally well positioned to become a dedicated platform able to concurrently reach a variety of neurological biomolecules and processes. Several studies have demonstrated that dimeric compounds exhibit significantly enhanced activity compared to monomeric compounds in multi-target anti-AD therapies. For instance, bis-tacrine (**2**) shows IC_50_ values of 0.81 nM, 5.66 nM, and 7.5 *μ*M for inhibiting AChE, BuChE and BACE1, respectively (Bolognesi et al., 2010). Similarly, bis-(arylvinyl)pyrazines (**3**) exhibit IC_50_ values of 17 nM and 13 nM for inhibiting Aβ aggregation and tau protein phosphorylation, respectively (Boländer et al., 2012). Moreover, while some bis-(2)-*β*-carbolines and bis-(9)-*β*-carbolines have been synthesized for the treatment of AD, such as **4** and **5** in Figure 1 (Rook et al., 2010; Zhao et al., 2018). This is noteworthy given that evidence suggests 7-substituted derivatives, such as **6** and **7**, can enhance activity against ACh, MAO, and A*β* aggregation to a certain extent (Du et al., 2023; Haider et al., 2018). Additionally, studies have shown that introducing various functional groups, such as allyl, propyl, and ethyl, at position 9 consistently enhances the anti-MAO activity (Beato et al., 2021).

Therefore, in this work, we propose the design of 9-substituted bis-(7)-harmine derivatives as multitarget-directed ligands (MTDLs) for management of AD. The potent inhibitory effects of these derivatives for *h*AChE, *h*BuChE, *h*MAO-B, *h*MAO-B, and A*β* aggregation were evaluated. Then the most promising derivatives were further evaluated for *in vitro* cytotoxic effects and their protective effects against amyloid-*β*-induced neurotoxicity, their mechanism of action through molecular docking studies, and their basic metabolic properties via ADMET computer predictions.

## 2 Results and discussion

### 2.1 Synthesis

**SCHEME 1:**
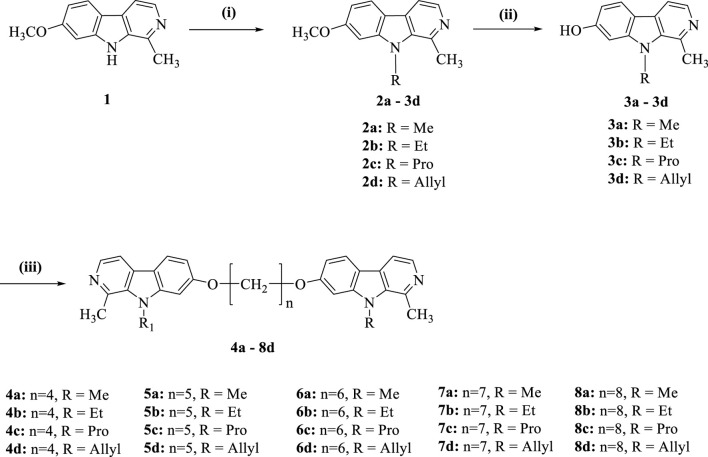
Synthesis of compounds **4a**−**8d**. Reagents and conditions: (i) NaH, RBr, DMF, rt.; (ii) AcOH, HBr, reflux; (iii) Cs_2_CO_3_, (CH_2_)_n_Br_2_, DMF.

In the present investigation, we aimed to synthesize a series of bis(7)-harmine derivatives **4a**–**8d** by connecting two 9-substituted harmine units through an ether linkage (Scheme 1). The synthesis commenced with harmine **1**, which was subjected to treatment with bromo-hydrocarbon and NaH in anhydrous DMF under conditions of 40°C to yield the 9-substituted harmine derivatives **2a**–**2d**. Subsequently, **2a**–**2d** were refluxed in AcOH and HBr for demethylation, resulting in the formation of **3a**–**3d**. Finally, the symmetrical dimers **4a**–**8d** were prepared by reacting different dibromo-alkanes with **3a**–**3d** in acetone using a catalytic amount of KCO_3_ (Du et al., 2023).

### 2.2 Biological evaluation

With a series of derivatives synthesized, we next conducted screenings against *h*AChE (1 *μ*M), *h*BuChE (1 *μ*M), *h*MAO-A (1 *μ*M), *h*MAO-B (1 *μ*M), and A*β*_1−42_ aggregation (20 μM) for all compounds, followed by determination of IC_50_ values for those demonstrating more than 50% inhibitory activity in the primary screening (Table 1).

**TABLE 1:** Inhibition of cholinesterase, monoamine oxidases and A*β*_1−42_ aggregation by the synthesized compounds.

Compound	IC_50_ (nM)^a^ for cholinesterase			IC_50_ (nM) for monoamine oxidases			IC_50_ (*μ*M) for A*β*_1−42_ ^c^
	*h*AChE	*h*BuChE	S.I. ^b^	*h*MAO-A	*h*MAO-B	S.I. ^b^	
4a	36.3%	30.5%	n.d ^d^	41.5%	13.8%	n.d	39.3%
4b	101.3 ± 6.8	36.7%	>9.9	685.6 ± 43.5	28.6%	>1.4	27.6%
4c	68 ± 5.1	694.4 ± 43.2	10.2	48.6%	17.8%	n.d	33.2%
4d	45.6%	32.9%	n.d	49.3%	30.3%	n.d	35.5%
5*a*	827.2 ± 41.5	256.3 ± 15.7	0.3	240.7 ± 13.8	25.6%	>4.1	40.6%
5*b*	115.4 ± 7.36	928.2 ± 62.1	8.1	42.4%	33.7%	n.d	31.8%
5*c*	73.8 ± 4.28	45.6%	>13.7	39.5%	27.5%	n.d	29.4%
5*d*	288.4 ± 21.3	41.2%	>3.4	41.1%	29.8%	n.d	43.6%
6a	493.3 ± 30.3	331.4 ± 21.5	0.7	122.8 ± 7.2	862.5 ± 61.3	7.1	19.8 ± 1.1
6b	652.4 ± 35.8	86.8 ± 5.3	0.1	893.1 ± 59.3	41.2%	>1.1	40.2%
6c	82.1 ± 6.9	994.4 ± 73.4	12.1	21.0 ± 1.18	529.4 ± 38.7	25.2	35.8%
6d	54.3 ± 3.6	102 ± 4.6	1.9	402 ± 23.7	189.3 ± 13.6	0.5	14.1 ± 0.5
7a	225.8 ± 13.4	37.3%	>4.4	7.2 ± 0.8	281.4 ± 24.5	40.1	6.3 ± 0.2
7b	385.7 ± 26.8	36.5%	>2.6	149.6 ± 12.4	79.8 ± 4.9	0.5	29.6%
7c	39.2 ± 2.9	44.8%	>25.6	9.1 ± 0.6	34.1%	>111.1	13.3 ± 0.6
7d	895.5 ± 65.4	23.7 ± 1.3	<0.02	74.6 ± 5.2	12.6 ± 0.76	0.2	17.1 ± 0.6
8a	42.2%	39.1%	n.d	95.2 ± 4.9	954.4 ± 49.8	10.0	16.5 ± 0.4
8b	323.6 ± 20.5	855.2 ± 48.8	2.7	66.5 ± 4.1	758.2 ± 52.6	11.5	18.7 ± 0.9
8c	158.1 ± 11.6	43.4%	>6.3	16.4 ± 1.1	152.6 ± 8.3	9.5	19.3 ± 1.0
8d	963.2 ± 69.5	39.8%	>1.0	25.7 ± 1.9	335.1 ± 13.8	111.7	9.2 ± 0.4
Harmine	30.9%	23.5%	n.d.	422.6 ± 20.8	23.5%	>2.3	31.3%
Tacrine	76.6 ± 4.3	n.d	>56.1	n.d	n.d	n.d	n.d
Resveratrol	n.d	n.d	n.d	n.d	n.d	n.d	11.5 ± 0.5
Rasagiline	n.d	n.d	n.d	n.d	73.6 ± 3.9	<0.07	n.d

^a^ Mean from 3-5 different assays (Mean ± SD).

^b^ S.I: Selectivity index. IC_50_(*h*BuChE)/IC_50_(*h*AChE) or IC_50_(*h*MAO-B)/IC_50_(*h*MAO-A).

^c^ The inhibition percent ratio of self-induced A*β*_1-42_ aggregation at a concentration of 20 *μ*M.

^d^ n.d. Not determined.

The Ellman’s assay was employed in this study to investigate the inhibitory potential of novel bis(7)-harmine derivatives on *h*AChE and *h*BuChE, along with tacrine as a reference compound (Ellman et al., 1961). As shown in Table 1, the majority of the tested target compounds exhibited superior inhibitory activity against *h*AChE compared to parent compound (harmine), with IC_50_ values at the nanomolar level. Furthermore, these compounds demonstrated remarkable selectivity towards *h*AChE over *h*BuChE. The initial analysis of the structure-activity relationship (SAR) suggested that both the nature of the 9-position substituents and the lengths of the carbon spacer significantly influenced their inhibitory activity against *h*AChE. Compounds featuring propyl substitution exhibited pronounced *h*AChE inhibition (IC_50_ in the range of 39.2–158.1 nM), suggesting a consistent superior inhibitory effect with propyl substituents at 9-position. However, as the carbon chain length increased, compounds containing propyl group showed diminished inhibition of *h*AChE. Moreover, when the substituent group at 9-position was methyl or allyl, *h*AChE inhibition initially increased and subsequently decreased with increasing spacer length. Conversely, compounds bearing an ethyl group demonstrated an initial decrease followed by an increase in inhibitory activity with elongation of the spacer. Notably, **4c**, **5c**, **6d**, and **7c** exhibited superior inhibitory activity against *h*AChE (IC_50_ = 68.0, 73.8, 54.3 and 39.2 nm, respectively), surpassing that of tacrine (positive control, IC_50_ = 76.6 nM). Additionally, **6a*−*6d** displayed dual inhibitory activities against both *h*AChE and *h*BuChE at nanomolar levels (IC_50_ values), indicating their potential as promising candidates for simultaneous targeting of the two enzymes in AD management. Compared with bis-(2)-*β*-carbolines and bis-(9)-*β*-carbolines, bis-(7)-*β*-carbolines exhibit comparable inhibitory activity against AChE. Notably, their inhibitory activity at the nanomolar level was significantly higher compared to monomer compounds at the micromolar level (Rook et al., 2010; Zhao et al., 2018). Therefore, bis-*β*-carbolines may be considered more promising candidates for anti-AD drug development compared to monovalent compounds.

The inhibitory potency against both *h*MAO-A and *h*MAO-B was investigated using recombinant human enzymes, with rasagiline serving as a positive control drug (Giovannuzzi et al., 2024). Generally, compounds containing a four- or five-carbon spacer exhibited limited inhibitory activity against both *h*MAO-A and *h*MAO-B (with IC_50_ values above 1 *μ*M), except for **4b** and **5a** which demonstrated inhibition of *h*MAO-A. In contrast, the twelve compounds featuring a six-, seven-, or eight-carbon spacer all exhibited significant inhibitory activity against *h*MAO-A, with the IC_50_ values ranging from 7.2 to 893.1 nM, while ten of these compounds also demonstrated notable inhibition towards *h*MAO-B (IC_50_ in the range of 12.6–954.4 nM). The findings suggested that the inhibition of *h*MAO by compounds was enhanced when employing a linker with a carbon spacer ranging from 6 to 8 atoms in length. Interestingly, **6d**, **7b**, and **7d** exhibited significant and selective inhibition of *h*MAO-B activity with IC_50_ values of 189.3 nM, 79.8 nM, and 12.6 nM, respectively. These values were comparable to or greater than that of rasagiline (IC_50_ = 73.6 nm).

The Thioflavin-T (ThT) fluorescence assay is a widely employed method for assessing the inhibitory efficacy of compounds on A*β*_1−42_ self-aggregation (Bian et al., 2024). In this study, bis(7)-harmine derivatives were examined for their inhibitory activity against A*β*_1−42_ self-aggregation, with resveratrol serving as a reference compound. As presented in Table 1, **4a−5d** featuring a four- or five-carbon spacer, exhibited relatively modest inhibitory activity against A*β*_1−42_ self-aggregation with IC_50_ values exceeding 20 *μ*M, indicating limited effectiveness in preventing amyloid plaque formation. Conversely, among the remaining twelve compounds with six-, seven-, or eight-carbon spacer, it was observed that nine of them demonstrated significant inhibition of A*β*_1−42_ aggregation. Interestingly, his trend exhibited a similar inhibitory pattern to that observed for MAO inhibitors. Moreover, **6d**, **7a**, **7c**, and **8d** demonstrated significant inhibition of A*β*_1−42_ aggregation (IC_50_ = 14.1, 6.3, 13.3, and 9.2 *μ*M, respectively), which were comparable to or greater than achieved by resveratrol (IC_50_ = 12.3 *μ*M). These results indicate that these compounds have the potential to effectively prevent or slow down the formation of a A*β*_1−42_ plaques in AD.

### 2.3 *In vitro* cytotoxicity

**FIGURE 2:**
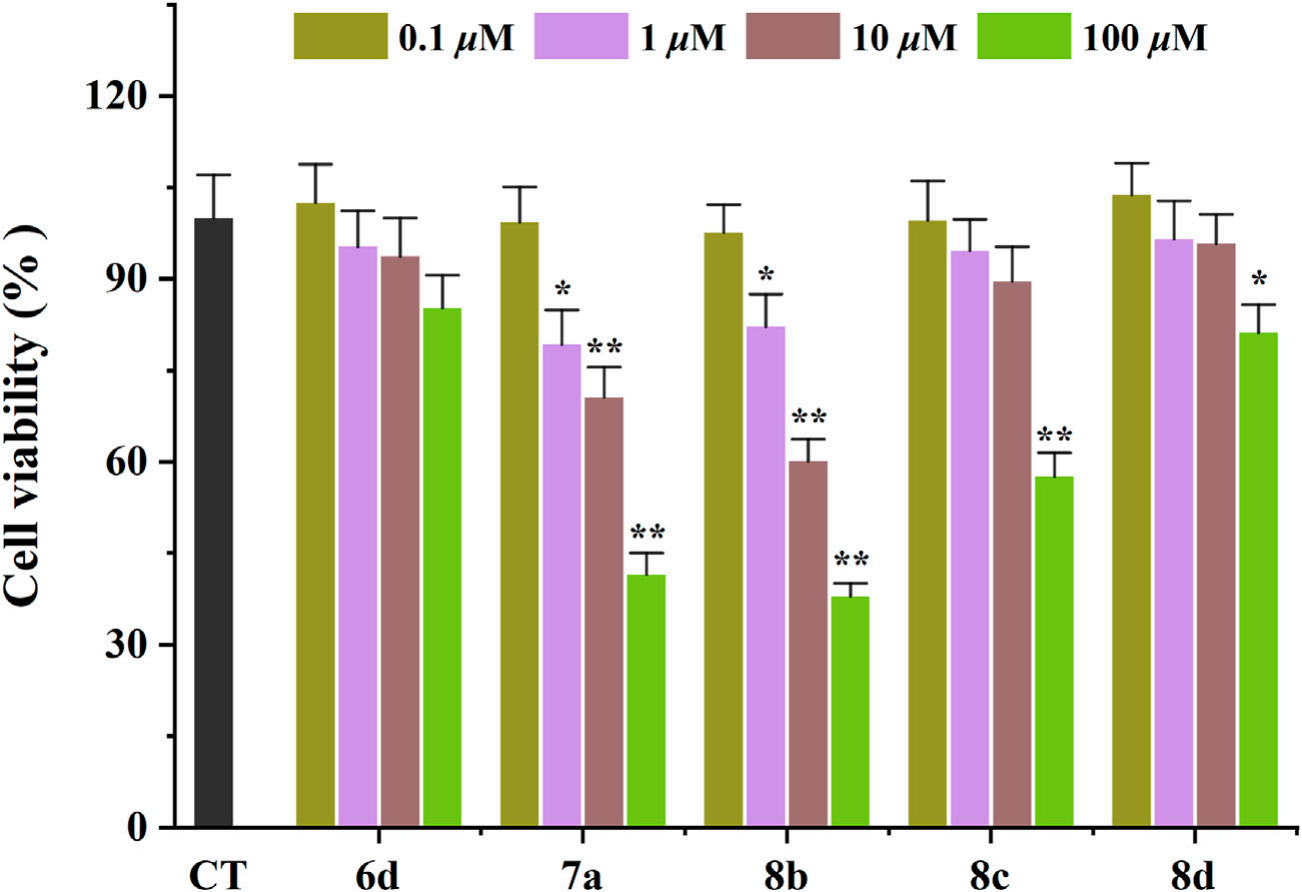
Neurotoxicity of compounds in SH-SY5Y cells. *p < 0.05, and **p < 0.01 vs*.* control group (untreated cells).

The toxicological properties of **6d**, **7a**, **8b**, **8c**, and **8d** were evaluated in the SHSY5Y human neuroblastoma cell model using an MTT reduction assay (Ellman et al., 1961). These compounds were selected based on their simultaneous inhibition of *h*AChE, *h*MAO-B, and A*β*_1−42_ self-aggregation. As depicted in Figure 2, **6d** exhibited no significant impact on cell viability within the concentration range of 0.1–100 *μ*M after a 48 h incubation period, thereby indicating its favorable safety profile. **8c** and **8d** demonstrated negligible cytotoxicity at concentrations ranging from 0.1 to 10 *μ*M; however, an evident decrease in SHSY5Y cell viability was observed when the concentration was increased to 10 *μ*M. These findings suggest that these compounds are considered safe at concentrations below or equal to 10 *μ*M. Unfortunately, **7a** and **8b** exerted a pronounced detrimental effect on SHSY5Y cell viability at a concentration of only 1 *μ*M. Consequently, due to their low toxicity profiles, **6d**, **8c**, and **8d** were consequently chosen for subsequent investigation.

### 2.4 Protection against A*β*_1−42_-induced neurotoxicity

**FIGURE 3:**
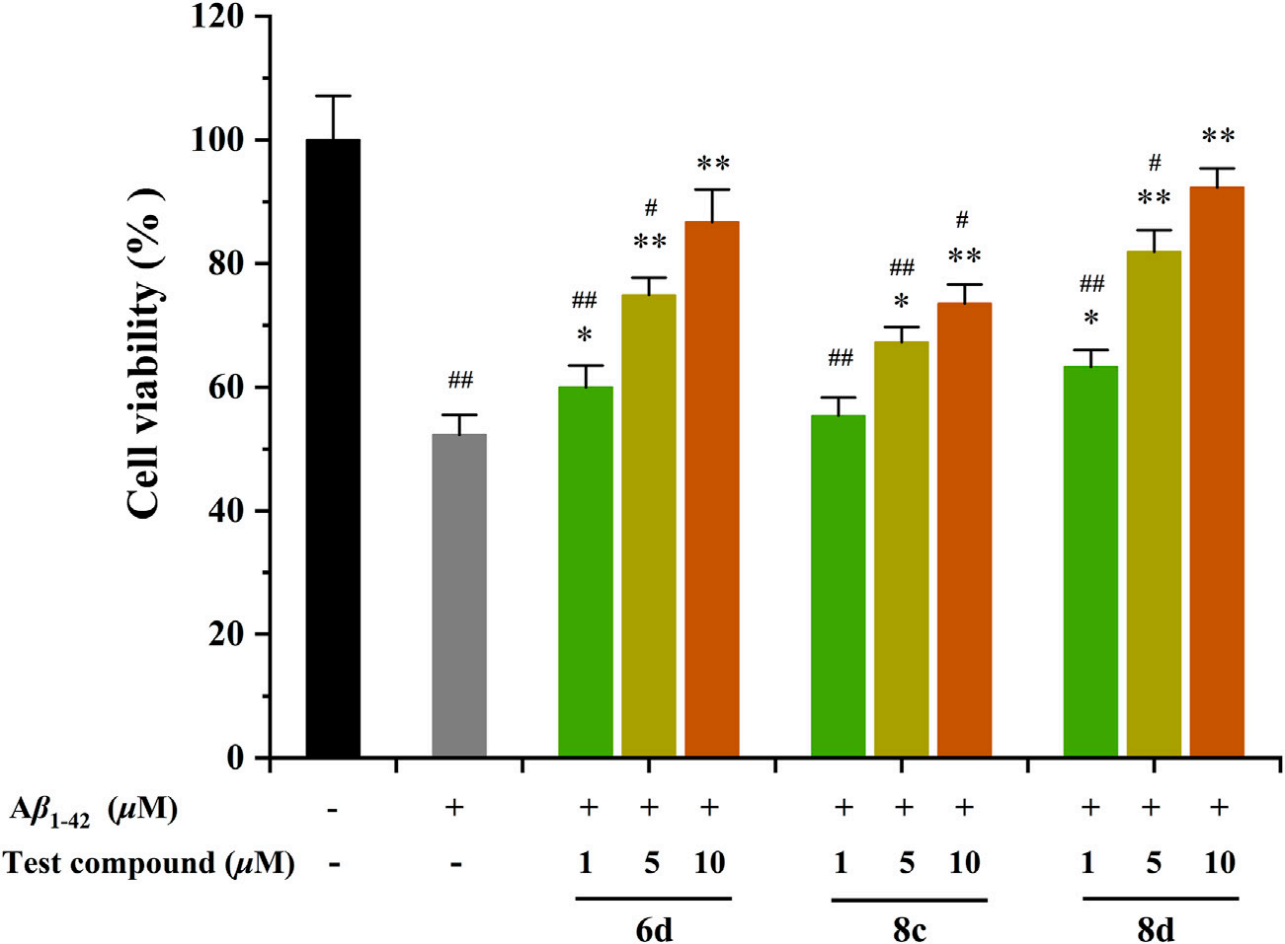
Neuroprotective effects of compounds **6d, 8c**, and **8d** against A*β*_1-42_-induced cell death in SH-SY5Y cells. ^#^ *p* < 0.05 and ^##^ *p* < 0.01 vs*.* control group (untreated cells); *p < 0.05 and **p < 0.001 vs*.* A*β*_1-42_-treated cells.

The protective effect of the test compounds against A*β*_1-42_-induced damage was assessed by monitoring MTT reduction in cells co-exposed to the compound and A*β*_1-42_ for 48 h (Figure 3) (Ellman et al., 1961). The **6d**, **8c**, and **8d** were tested at concentrations of 1, 5, and 10 *μ*M. The viability of SHSY5Y neuroblastoma cells was significantly reduced by 48.7% after treatment with A*β*_1−42_. Surprisingly, the three compounds exhibited a notable neuroprotective efficacy within the concentration range of 5–10 *μ*M. Specifically, **6d** and **8d** exhibited remarkable efficacy, demonstrating significant cellular recovery even at a minimal concentration of only 1 *μ*M. Moreover, the two compounds exhibited nearly complete protection against neuronal damage when their concentration reached 10 *μ*M. The results demonstrate that the tested compounds exhibit the potential to alleviate A*β*-related toxicity, thereby presenting promising therapeutic prospects for AD. Therefore, it is imperative to elucidate the underlying mechanisms responsible for these protective effects.

### 2.5 Docking studies

**FIGURE 4:**
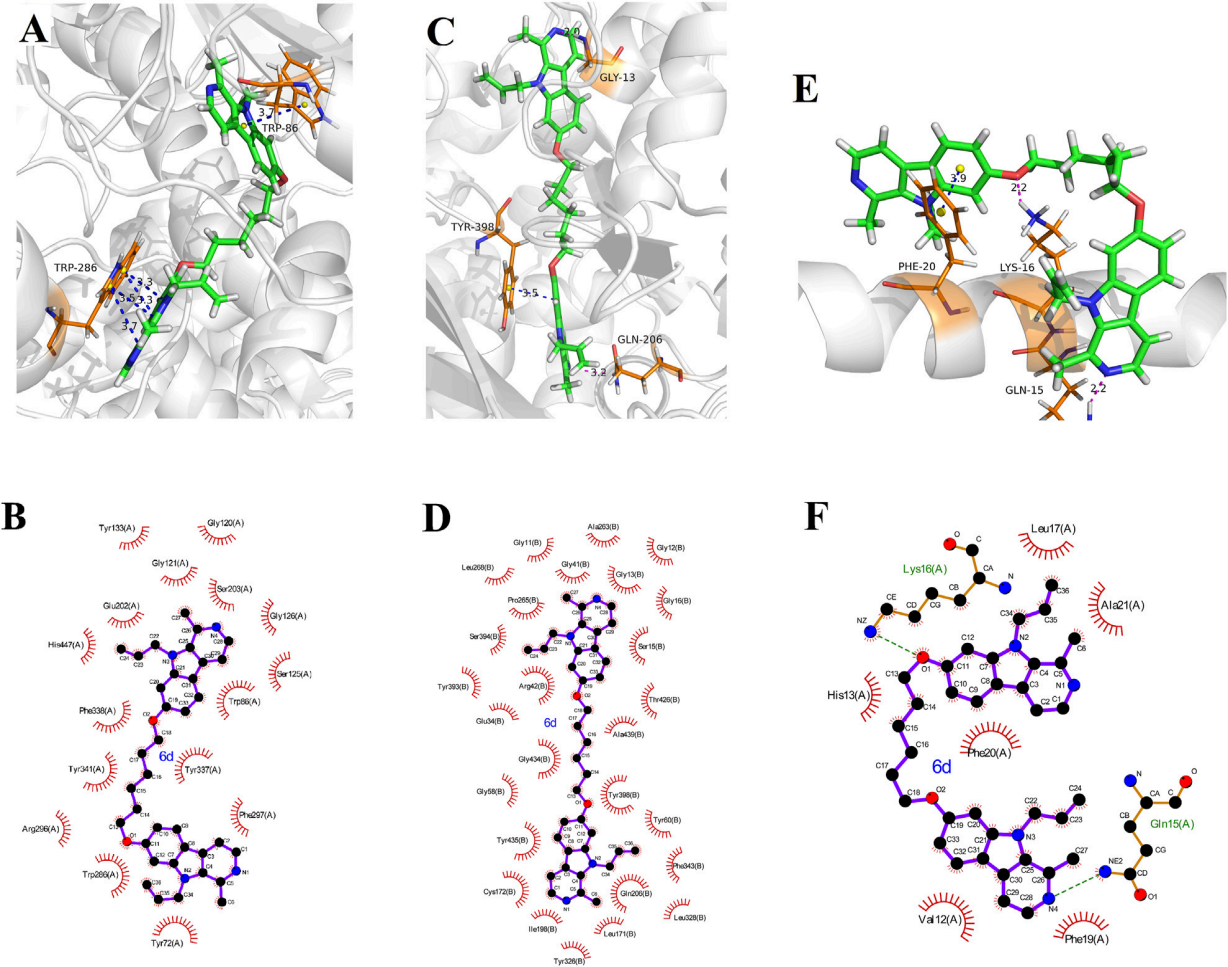
Molecule docking results: **(A, B)** were 3D and 2D docking models of compound 6d with AChE (4EY7); **(C, D)** were 3D and 2D docking models of compound 6d with *h*MAO-B (2V60); **(E, F)** were 3D and 2D docking models of compound 6d with A*β*_1-42_ (1IYT). The blue dashed lines stand for *π*-*π* stacking, and the magentas dashed lines represent hydrogen bond.

To elucidate the binding mechanism of bis(7)-harmine derivatives towards their target enzymes, we investigated the binding interactions of one of the most potent compound, **6d**, with *h*AChE (PDB code: 4EY7), *h*MAO-B (PDB code: 2V60) and A*β*_1-42_ (PDB code: 1IYT), respectively (Binda et al., 2007; Cheung et al., 2012; Crescenzi et al., 2002). The Sybyl−X 2.0 software was used to predict the most energetically stable configurations of the ligand-target complexes (Figures 4A–F).

The 3D docking model (Figure 4A) illustrates a π−π stacking interaction between the pyrrole ring of harmine and residue Trp86 at a distance of 3.8 Å, facilitating binding to the CAS of AChE. Furthermore, another harmine group binds to the PAS of AChE through four π−π interactions with residue Trp286 at distances of 3.3 Å, 3.3 Å, 3.5 Å, and 3.7 Å, respectively. This observation indicates that these derivatives possess the ability to simultaneously interact with both the CAS and PAS domains as intended in our design objective. Additionally, hydrophobic contacts were observed between **6** and residues Tyr72, Trp86, Gly120, Gly121, Ser125, Gly126, Tyr133, Glu202, Ser203, Trp286, Arg296, Phe297, Tyr337, Phe338, Tyr341, and His447 in the 2D docking model (Figure 4B). These favorable interactions are likely to contribute to the enhanced inhibitory efficacy of ligands. Therefore, the enhancement of electron-donating groups on the harmine structure and/or increased hydrophobicity of the derivatives could potentially enhance *h*AChE inhibitory activity as supported by structure-activity relationship studies.

The Figure 4C reveals intriguing hydrogen bonds formed by N atoms of pyridines in two harmine fragments with Gly13 and Gln206 at distances of 2.0 and 3.2 Å, respectively. Additionally, one of the harmine fragments engages in a π−π stacking interaction with residue Try398 at a distance of 3.5 Å. Moreover, we have identified twenty-seven residues (including Gly12, Gly13, Ser15 etc., as shown in Figure 4D) that participate in hydrophobic interactions potentially enhancing the *h*MAO-B inhibition efficacy.

In Figure 4E, it is noteworthy that residue Phe20 of A*β*_1-42_ forms a π−π stacking interaction with the benzene ring in one of the harmine fragments at a distance of 3.9 Å. Additionally, the N atom of pyridine in other harmine moieties interacts with residue Gln15 through intermolecular hydrogen bonding at a distance of 2.2 Å. Furthermore, the O atom at 7-position of the harmine fragment engages in an intermolecular hydrogen bonding interaction with residue Lys16 at a distance of 2.2 Å. Moreover, hydrophobic interactions between **6d** and residues (such as Val12, His13, Leu17, Phe19, Phe20, and Ala21) from Figure 4F are observed. These observed interactions provide potential mechanisms for the high inhibitory activity against A*β*_1−42_ aggregation exhibited by this ligand.

### 2.6 ADMET prediction

The absorption, distribution, metabolism, excretion, and toxicity properties (ADMET) of the most potent derivatives **6d**, **8c**, and **8d** were evaluated using *in silico* ADMET prediction methods (Xiong et al., 2021; Banerjee et al., 2018). Tacrine, resveratrol, and rasagiline were utilized as reference drugs for comparative analysis. As presented in Table 2, the *in vitro* permeability of CaCo-2 (Caucasian colon adenocarcinoma) cells fell within the intermediate range (31.6–34.7 nm/s), demonstrating a significantly higher value compared to resveratrol (5.2 nm/s) and closely resembling that of tacrine (25.9 nm/s). Moderate values of *invitro* MDCK (Madin–Darby canine kidney) cells were observed (51.7–65.5 nm/s), indicating an acceptable range of permeability for these derivatives. One crucial aspect assessed was high intestinal absorption (HIA). It was found that all target derivatives exhibited HIA values ranging from 98.3% to 98.4%, which are remarkably similar to those observed for reference drugs like tacrine (96.5%) and rasagiline (100%). This suggests that these derivatives have a high potential for efficient absorption through the intestines.

**TABLE 2:** ADMET properties predicted *in silico* for some active compounds, tacrine, resveratrol and rasagiline.

Compound	Absorption			Distribution			Metabolism					Excretion	Toxicity	
	Caco−2^a^	MDCK^b^	HIA^c^	PPB^d^	BBB^e^	VD^f^	CYP1A2 inhibitor	CYP2C19 inhibitor	CYP2C9 inhibitor	CYP2D6 inhibitor	CYP3A4 inhibitor	CL^g^	LD_50_	Toxicity class
6d	34.7	51.7	98.4	90.6	0.3	1.1	No	No	No	Yes	Yes	6.4	1,000	4
8c	31.6	65.5	98.3	91.3	0.6	4.6	No	No	No	Yes	No	5.6	1,000	4
8d	33.4	64.0	98.3	91.2	0.4	1.2	No	No	No	Yes	Yes	5.9	1,000	4
Tacrine	25.9	38.5	96.5	63.8	0.9	1.6	Yes	No	No	Yes	Yes	3.6	40	2
Resveratrol	5.2	76.7	88.5	100	1.7	0.7	Yes	No	Yes	No	Yes	14.4	1,560	4
Rasagiline	50.5	46.58	100	64.9	6.34	3.1	No	No	No	No	No	5.6	2000	4

^a^ Caco-2, cell permeability (nm/sec), a poor permeation for values <25 and high permeation for values >500.

^b^ MDCK, cell permeability (nm/sec), poor: <25, high: >500.

^c^ Human intestinal absorption (%), 70–100%.

^d^ Plasma protein binding (%), >90%.

^e^ Blood–brain barrier penetration (C. brain/C. blood), >0.4.

^f^ Volume of distribution (L/Kg), 0.04–20.

^g^ Clearance (mL/min/kg), poor: <5, moderate: 5–15, high: >500.

The predicted PPB (Plasma protein binding) values for the tested derivatives, ranging from 90.6% to 91.3%, were found to be comparable to that of resveratrol (100%). This suggests that these derivatives exhibit a high affinity for plasma proteins, potentially enhancing their stability and prolonging their therapeutic effects in the bloodstream. Furthermore, the calculated BBB (blood−brain barrier) permeabilities of **6d**, **8c**, and **8d** were determined as 0.3, 0.6 and 0.4 respectively, which closely approximate the required value of 0.4. These results imply favorable characteristics of these compounds in terms of crossing the blood-brain barrier and reaching target sites within the CNS effectively. Additionally, all three derivatives exhibited predicted VD (volumes of distribution) falling within an optimal range between 0.04 and 20 L/kg, suggesting a well-balanced distribution throughout various tissues in the body after administration.

The metabolism of drugs in the body is a complex process that greatly influences their concentration in the bloodstream. To better understand how certain drugs interact with enzymes involved in drug metabolism, such as cytochrome P450 isoforms, researchers often rely on *in silico* studies. In this study, the focus was on predicted inhibitors targeting specific cytochrome P450 isoforms, such as CYP1A2, CYP2C19, CYP2C9, CYP2D6, and CYP3A4. These isoforms are known to play crucial roles in drug metabolism and can significantly affect the efficacy and safety of medications. Among the three tested derivatives (**6d**, **8c**, and **8d**), they exhibited a similar profile to tacrine by lacking inhibitory effects on the CYP2C19 and CYP2C9 isoforms while demonstrating inhibitory activity towards the CYP2D6 and CYP3A4 isoforms. Notably, similar to rasagiline, none of these derivatives displayed any inhibitory potential against the CYP1A2 isoform.

Excretion plays a pivotal role in pharmacokinetics as it governs the efficient elimination of drug derivatives from the body. To comprehend this process, predictions were made based on their CL (clearance rate). It is noteworthy that all three derivatives exhibited moderate clearance rates ranging from 5.6 to 6.4 mL/min/kg.

The toxicity properties predicted for **6d**, **8c**, and **8d** have provided valuable insights into their safety profiles. These compounds exhibited LD_50_ values (1,000 mg/kg) that were 25-times as high as tacrine (40 mg/kg), but significantly lower than those of resveratrol (1,560 mg/kg) and rasagiline (2000 mg/kg). All tested compounds were classified at level 4 for toxicity, similar to the control drugs (resveratrol and rasagiline). Based on these predictions, it can be inferred that the derivatives possess low toxicity levels and exhibit favorable safety profiles.

The majority of predicted ADMET property parameters in **6d**, **8c**, and **8d** fall within the optimal range for favorable pharmacokinetic characteristics. Consequently, these compounds demonstrate promising potential as lead candidates for Alzheimer’s disease treatment. However, further investigation is warranted to assess their *in vivo* activity.

## 3 Conclusion

In this study, a series of bis(7)-harmine derivatives were designed and synthesized with the aim of developing effective multi-target ligands for the treatment of Alzheimer’s disease (AD). The researchers evaluated the biological profile of these derivatives against various AD-related targets, including cholinesterases, monoamine oxidase, and A*β* aggregation. Out of the 20 compounds investigated, seventeen showed significant inhibitory activity against *h*AChE at nanomolar levels. This suggests that these compounds have the potential to effectively inhibit acetylcholinesterase (AChE), an enzyme involved in the breakdown of acetylcholine in the brain. Furthermore, ten compounds demonstrated nanomolar inhibitory activity against *h*MAO-B. MAO-B is another target implicated in AD pathology. Inhibiting MAO-B can help prevent the breakdown of neurotransmitters such as dopamine and serotonin, which are important for maintaining proper brain function. Additionally, nine derivatives displayed noteworthy inhibition of A*β*_1−42_ aggregation. A*β* aggregation is a hallmark feature observed in AD patients' brains. By preventing or reducing A*β* aggregation, these compounds may potentially slow down disease progression. Further investigation revealed that three specific compounds **6d**, **8c**, and **8d** exhibited significant neuroprotective activity against A*β*_1−42_-induced damage in SH-SY5Y cells while showing low toxicity towards neuroblastoma cells (SH-SY5Y). This indicates their potential as therapeutic agents for protecting neurons from harmful effects associated with amyloid-beta accumulation. Molecular modeling studies provided valuable insights into the mechanism of action of **6**. It was found that the binding processes were primarily driven by hydrophobic interactions, hydrogen bonding, and π-π stacking interactions with important residues of *h*AChE, *h*MAO-B and A*β*. Furthermore, ADMET prediction results indicated favorable characteristics for **6d**, **8c**, and **8d** as potential drugs for AD management. These compounds showed promising pharmacokinetic properties such as good absorption, distribution within the brain tissue, metabolism without generating toxic metabolites or exhibiting significant drug-drug interactions. Overall, these findings provided strong evidence supporting the further development of bis(7)-harmine derivatives as lead compounds in research towards potential anti-AD drug candidates.

## 4 Materials and methods

### 4.1 General remarks

The solvents, chemicals, and reagents were procured from commercial suppliers without undergoing additional purification steps. Thin layer chromatography (TLC) was utilized to monitor the progress of the reactions. Silica gel (200–300 mesh) obtained from Qingdao Marine Chemical Company was employed for flash chromatography purification. The Bruker Avance III instrument operating at 500 MHz for ^1^H and 125 MHz for ^13^C was used to acquire the 1H NMR and 13C NMR spectra. Chemical shifts were reported in parts per million (ppm, δ), with CDCl_3_ serving as the solvent and tetramethylsilane (TMS) acting as an internal standard. Singlet (s), doublet (d), triplet (t), doublet of doublet (dd), or multiplet (m) designations were assigned based on multiplicities observed. Agilent 6520 Q−TOF LC/MS was employed to obtain high resolution mass spectra (HRMS).

### 4.2 General procedure for the synthesis of bis(7)-harmine derivatives 4a–8d

The synthesis of compounds was carried out using a previously reported method (Du et al., 2023). To a solution of harmine **1** (5 mmol) and bromo-hydrocarbon (7.5 mmol) in anhydrous DMF (100 mL), the mixture was stirred at 0°C for 5 min. Subsequently, NaH (10 mmol) was added to the suspension with stirring at 40°C for 8 h under TLC monitoring. Upon completion of the reaction, the solvent was gradually added to a 50 mL ice-water solution and left at 5°C for 12 h. After filtration, the solid underwent recrystallization with acetone, resulting in the formation of 9-substituted harmine derivatives **2a**–**2d**. Subsequently, **2a**–**2d** (3 mmol) were refluxed in a mixture of AcOH (30 mL) and HBr (*v*: *v* = 1: 1) for 24 h. After completion of the reaction, the solvents were evaporated under reduced pressure followed by addition of distilled water to precipitate the resulting solid. The obtained solid (**3a**–**3d**) was used in subsequent steps without further purification. Different dibromo-alkanes (1 mmol) were added to a solution of **3a**–**3d** (2 mmol) in acetone (20 mL). Then KCO_3_ (8 mmol) and a small amount of KI were added to the mixture which was heated to reflux for 12 h. Afterward, the mixture was concentrated *in vacuo* and washed with water three times. Finally, the product was purified by column chromatography to yield **4a**–**8d.**

#### 4.2.1 7-Methoxy-1,9-dimethyl-9*H*-pyrido [3,4-b]indole (**2a**)

White solid; yield 91%; ^1^H NMR (500 MHz Chloroform-*d*) 8.24 (d, *J* = 5.2 Hz, 1H), 7.92 (d, *J* = 8.6 Hz, 1H), 7.68 (d, *J* = 5.1 Hz, 1H), 6.86 (dd, *J* = 6.5, 1.7 Hz, 2 H), 6.78 (d, *J* = 1.7 Hz, 1H), 4.00 (s, 3H), 3.93 (s, 3H), 3.02 (s, 3H); ^13^C NMR (125 MHz, Chloroform-*d*) *δ* 160.9, 143.6, 141.0, 138.2, 136.0, 129.0, 122.3, 114.9, 112.2, 108.9, 92.9, 55.7, 32.2, and 23.5.

#### 4.2.2 9-Ethyl-7-methoxy-1-methyl-9*H*-pyrido [3,4-*b*]indole (**2b**)

White solid; yield 84%; ^1^H NMR (500 MHz Chloroform-*d*) 8.27 (d, *J* = 5.2 Hz, 1 H), 7.97 (d, *J* = 8.5 Hz, 1H), 7.72 (d, *J* = 5.1 Hz, 1H), 6.88 (dd, *J* = 1.8, 8.5 Hz, 1H), 6.86 (d, *J* = 1.8 Hz, 1H), 4.54 (q, *J* = 7.1 Hz, 2H), 3.95 (s, 3H), 3.02 (s, 3H), 1.44 (t, *J* = 7.1 Hz, 3H); ^13^C NMR (125 MHz, Chloroform-*d*) *δ* 160.9, 142.7, 140.5, 138.2, 135.1, 129.4, 122.4, 115.3, 112.3, 108.8, 93.1, 55.7, 39.5, 23.2, 15.5.

#### 4.2.3 7-Methoxy-1-methyl-9-propyl-9*H*-pyrido [3,4-*b*]indole (**2c**)

White solid; yield 75%; ^1^H NMR (500 MHz Chloroform-*d*) 8.27 (d, *J* = 5.2 Hz, 1 H), 7.95 (d, *J* = 8.5 Hz, 1H), 7.71 (d, *J* = 5.1 Hz, 1H), 6.87 (dd, *J* = 1.8, 8.5 Hz, 1H), 6.83 (d, *J* = 1.8 Hz, 1H), 4.39 (t, *J* = 7.7 Hz, 1H), 3.93 (s, 3H), 2.99 (s, 3H), 1.88–1.78 (m, 2 H), 1.00 (t, *J* = 7.4 Hz, 3H); ^13^C NMR (125 MHz, Chloroform-*d*) *δ* 160.8, 143.1, 140.6, 138.3, 135.4, 129.3, 122.3, 115.2, 112.2, 108.6, 93.5, 55.7, 46.3, 23.9, 23.4, and 11.3.

#### 4.2.4 9-Allyl-7-methoxy-1-methyl-9*H*-pyrido [3,4-*b*]indole (**2d**)

Light yellow; yield 87%; ^1^H NMR (500 MHz, Chloroform-*d*) *δ* 8.27 (d, *J* = 5.2 Hz, 1H), 7.95 (d, *J* = 8.5 Hz, 1H), 7.71 (d, *J* = 5.2 Hz, 1H), 6.87 (dd, *J* = 8.6, 2.2 Hz, 1H), 6.84 (d, *J* = 2.2 Hz, 1H), 6.30–6.22 (m, 1H), 5.30 (dt, *J* = 3.9, 2.2 Hz, 2H), 5.29 (dd, *J* = 10.5, 2.2 Hz, 1H), 4.80 (dd, *J* = 17.3, 2.0 Hz, 1H), 3.94 (s, 3H), 3.00 (s, 3H); ^13^C NMR (125 MHz, Chloroform-*d*) *δ* 160.8, 143.1, 140.6, 138.3, 135.3, 133.2, 129.3, 122.3, 115.2, 114.1, 112.2, 108.5, 93.5, 47.5, 44.7, and 23.5.

#### 4.2.5 1,4-bis ((1,9-dimethyl-9*H*-pyrido [3,4-*b*]indol-7-yl)oxy)butane (**4a**)

White solid; yield 55%; ^1^H NMR (500 MHz, Chloroform-*d*) *δ* 8.26 (d, *J* = 5.3 Hz, 2H), 7.96 (d, *J* = 8.4 Hz, 2H), 7.75 (d, *J* = 5.3 Hz, 2H), 6.91 (dd, *J* = 8.6, 2.0 Hz, 2H), 6.85 (d, *J* = 2.0 Hz, 2H), 4.20 (t, *J* = 6.2 Hz, 4H), 4.01 (s, 6H), 3.01 (s, 6H), 1.98–1.92 (m, 4H); ^13^C NMR (125 MHz, Chloroform-*d*) *δ* 160.4, 143.7, 140.4, 136.9, 135.7, 129.4, 122.1, 114.3, 112.2, 109.6, 93.5, 68.0, 32.3, 28.7, and 22.4; ESI–MS m/z Calcd for C_30_H_30_N_4_O_2_ [M + H]^+^ 479.2402, found 479.2463.

#### 4.2.6 1,4-bis ((9-ethyl-1-methyl-9*H*-pyrido [3,4-*b*]indol-7-yl)oxy)butane (**4b**)

White solid; yield 59%; 1H NMR (500 MHz, Chloroform-*d*) *δ* 8.24 (d, *J* = 5.1 Hz, 2H), 7.94 (d, *J* = 8.5 Hz, 2H), 7.70 (d, *J* = 5.1 Hz, 2H), 6.86 (dd, *J* = 8.5, 2.1 Hz, 2H), 6.84 (d, *J* = 2.0 Hz, 2H), 4.40 (q, *J* = 7.0 Hz, 4H), 4.15 (t, *J* = 6.2 Hz, 4H), 2.99 (s, 6H), 1.97–2.02 (m, 4H), 1.43 (t, *J* = 7.1 Hz, 6H); ^13^C NMR (125 MHz, Chloroform-*d*) *δ* 160.6, 143.2, 141.2, 138.5, 135.8, 129.7, 122.8, 115.6, 112.7, 109.5, 94.4, 68.4, 44.8, 28.6, 22.5, and 14.9; ESI–MS m/z Calcd for C_32_H_34_N_4_O_2_ [M + H]^+^ 507.2715, found 507.2733.

#### 4.2.7 1,4-bis ((1-methyl-9-propyl-9*H*-pyrido [3,4-*b*]indol-7-yl)oxy)butane (**4c**)

White solid; yield 54%; ^1^H NMR (500 MHz, Chloroform-*d*) *δ* 8.27 (d, *J* = 5.2 Hz, 2H), 7.94 (d, *J* = 8.5 Hz, 2H), 7.71 (d, *J* = 5.1 Hz, 2H), 6.83–6.87 (m, 4H), 4.38 (t, *J* = 7.7 Hz, 4H), 4.16 (d, *J* = 5.8 Hz, 4H), 3.00 (s, 6H), 1.98–2.02 (m, 4H), 1.88–1.91 (m, 4H), 0.99 (t, *J* = 7.3 Hz, 6H); ^13^C NMR (125 MHz, Chloroform-*d*) *δ* 160.2, 143.3, 140.1, 137.9, 135.0, 129.2, 122.1, 115.3, 112.2, 109.5, 93.5, 68.3, 56.2, 28.5, 24.2, 22.4, and 11.5; ESI–MS m/z Calcd for C_34_H_38_N_4_O_2_ [M + H]^+^ 535.3028, found 535.3040.

#### 4.2.8 1,4-bis ((9-allyl-1-methyl-9*H*-pyrido [3,4-*b*]indol-7-yl)oxy)butane (**4d**)

White solid; yield 46%; ^1^H NMR (500 MHz, Chloroform-*d*) *δ* 8.30 (d, *J* = 5.2 Hz, 2H), 8.00 (d, *J* = 8.6 Hz, 2H), 7.76 (d, *J* = 5.2 Hz, 2H), 6.90 (dd, *J* = 2.0, 8.5 Hz, 2H), 6.87 (d, *J* = 1.9 Hz, 2H), 6.30–6.22 (m, 2H), 5.34–5.26 (m, 6H), 4.84–4.78 (m, 2H), 4.16 (t, *J* = 6.2 Hz, 4H), 2.90 (s, 6H), 1.98–2.01 (m, 4H); ^13^C NMR (125 MHz, Chloroform-*d*) *δ* 160.6 143.2, 140.5, 138.4, 135.4, 132.8, 129.3, 122.4, 112.1, 115.4, 114.0, 112.1, 109.4, 94.3, 67.9, 46.2, 23.5, and 15.3; ESI–MS m/z Calcd for C_34_H_34_N_4_O_2_ [M + H]^+^ 531.2715, found 531.2747.

#### 4.2.9 1,5-bis ((1,9-dimethyl-9*H*-pyrido [3,4-*b*]indol-7-yl)oxy)pentane (**5a**)

White solid; yield 43%; ^1^H NMR (500 MHz, Chloroform-*d*) *δ* 8.27 (d, *J* = 5.3 Hz, 2H), 7.96 (d, *J* = 8.6 Hz, 2H), 7.75 (d, *J* = 5.3 Hz, 2H), 6.91 (dd, *J* = 8.6, 2.2 Hz, 2H), 6.85 (d, *J* = 2.1 Hz, 2H), 4.18 (t, *J* = 6.3 Hz, 4H), 4.02 (s, 6H), 2.99 (s, 6H), 2.08–1.94 (m, 4H), 1.90–1.81 (m, 2H); ^13^C NMR (125 MHz, Chloroform-*d*) *δ* 160.4, 143.8, 140.4, 136.9, 135.7, 129.3, 122.1, 114.3, 112.2, 109.5, 93.4, 68.0, 31.9, 29.8, 22.5, and 22.0; ESI–MS m/z Calcd for C_31_H_32_N_4_O_2_ [M + H]^+^ 493.2559, found 493.2598.

#### 4.2.10 1,5-bis ((9-ethyl-1-methyl-9*H*-pyrido [3,4-*b*]indol-7-yl)oxy)pentane (**5b**)

White solid; yield 52%; ^1^H NMR (500 MHz, Chloroform-*d*) *δ* 8.27 (d, *J* = 5.2 Hz, 2H), 7.95 (d, *J* = 8.5 Hz, 2H), 7.72 (d, *J* = 5.2 Hz, 2H), 6.95–6.87 (m, 2H), 6.86 (d, *J* = 2.4 Hz, 2H), 4.50 (q, *J* = 7.1 Hz, 4H), 4.16 (t, *J* = 6.2 Hz, 4H), 3.01 (s, 6H), 2.03–1.96 (m, 4H), 1.84–1.76 (m, 2H), 1.42 (t, *J* = 7.1 Hz, 6H); ^13^C NMR (125 MHz, Chloroform-*d*) *δ* 160.4, 142.7, 140.5, 138.1, 135.1, 129. 5, 122.4, 115.3, 112.3, 109.1, 93.8, 68.3, 39.5, 29.2, 23.2, 22.9, and 15.5; ESI–MS m/z Calcd for C_33_H_36_N_4_O_2_ [M + H]^+^ 521.2872, found 521.2903.

#### 4.2.11 1,5-bis ((1-methyl-9-propyl-9*H*-pyrido [3,4-*b*]indol-7-yl)oxy)pentane (**5c**)

Light yellow solid; yield 47%; ^1^H NMR (500 MHz, Chloroform-*d*) *δ* 8.24 (2H, d, *J* = 5.2 Hz, 2H), 7.92 (2H, d, *J* = 8.4 Hz, 2H), 7.74 (2H, d, *J* = 5.2 Hz, 2H), 6.86 (dd, *J* = 8.6, 2.1 Hz, 2H, 2H), 6.82 (d, *J* = 2.1 Hz, 2H), 4.40 (d, *J* = 7.7 Hz, 1H), 4.15 (t, *J* = 6.0 Hz, 4H), 2.98 (s, 6H), 1.94–1.98 (m, 4H), 1.76–1.83 (m, 4H), 1.63–1.68 (m, 2H), 0.98 (t, *J* = 7.2 Hz, 6H); ^13^C–NMR (125 MHz, Chloroform-*d*) *δ* 160.7, 143.1, 140.4, 138.2, 135.3, 129.1, 122.2, 115.0, 112.1, 109.0, 93.8, 67.9, 56.6, 29.2, 25.4, 23.1, 21.2, and 11.6; ESI–MS m/z Calcd for C_35_H_40_N_4_O_2_ [M + H]^+^ 549.3185, found 549.3218.

#### 4.2.12 1,5-bis ((9-allyl-1-methyl-9*H*-pyrido [3,4-*b*]indol-7-yl)oxy)pentane (**5d**)

Light yellow solid; yield 61%; ^1^H NMR (500 MHz, Chloroform-*d*) *δ* 8.28 (d, *J* = 5.2 Hz, 2H), 7.99 (d, *J* = 8 .6 Hz, 2H), 7.75 (d, *J* = 5.2 Hz, 2H), 6.96 (dd, *J* = 2.0, 8.5 Hz, 2H), 6.90 (d, *J* = 2.0 Hz, 2H), 6.29–6.22 (m, 2H), 5.28 (dt, *J* = 4.0, 2.1 Hz, 4H), 5.26 (dd, *J* = 10.2, 2.1 Hz, 2H), 4.80 (dd, *J* = 16.8, 2.0 Hz, 2H), 4.15 (t, *J* = 6.0 Hz, 4H), 2.98 (s, 6H), 2.14–2.06 (m, 4H), 1.80–1.74 (m, 2H); ^13^C NMR (125 MHz, Chloroform-*d*) *δ* 160.5, 143.0, 142.3, 139.1, 135.5, 129.4, 127.3, 126.2, 122.4, 115.3, 112.5, 109.6, 95.8, 67.9, 48.3, 27.2, 21.5, and 15.4; ESI–MS m/z Calcd for C_35_H_36_N_4_O_2_ [M + H]^+^ 545.2872, found 545.2803.

#### 4.2.13 1,6-bis ((1,9-dimethyl-9*H*-pyrido [3,4-*b*]indol-7-yl)oxy)hexane (**6a**)

Yellow solid; yield 59%; ^1^H NMR (500 MHz, Chloroform-*d*) *δ* 8.26 (d, *J* = 5.2 Hz, 2H), 7.97 (d, *J* = 8.3 Hz, 2H), 7.76 (d, *J* = 5.2 Hz, 2H), 6.89–6.91 (m, 4H), 4.15 (t, *J* = 6.2 Hz, 4H), 4.05 (s, 6H), 2.99 (s, 6H), 2.02–1.98 (m, 4H), 1.72–1.64 (m, 4H); ^13^C–NMR (125 MHz, Chloroform -*d*) *δ* 160.4, 142.6, 139.8, 137.0, 134.9, 129.6, 122.1, 114.8, 112.3, 109.3, 93.8, 65.8, 39.1, 29.3, 24.9, and 22.4; ESI–MS m/z Calcd for C_32_H_34_N_4_O_2_ [M + H]^+^ 507.2715, found 507.2769.

#### 4.2.14 1,6-bis ((9-ethyl-1-methyl-9*H*-pyrido [3,4-*b*]indol-7-yl)oxy)hexane (**6b**)

White solid; yield 41%; ^1^H NMR (500 MHz, Chloroform-*d*) *δ* 8.26 (d, *J* = 5.3 Hz, 2H), 7.97 (d, *J* = 8.5 Hz, 2H), 7.76 (d, *J* = 5.3 Hz, 2H), 6.99–6.80 (m, 4H), 4.54 (q, *J* = 7.1 Hz, 4H), 4.15 (t, *J* = 6.4 Hz, 4H), 2.99 (s, 6H), 1.95 (t, *J* = 7.4 Hz, 4H), 1.76–1.69 (m, 4H), 1.44 (t, *J* = 7.1 Hz, 6H); ^13^C NMR (125 MHz, Chloroform-*d*) *δ* 160.5, 142.8, 140.0, 137.1, 134.9, 129.7, 122.3, 114.8, 112.3, 109.4, 93.6, 68.2, 39.9, 29.1, 25.8, 22.1, and 15.3; ESI–MS m/z Calcd for C_34_H_38_N_4_O_2_ [M + H]^+^ 535.3073, found 535.3098.

#### 4.2.15 1,6-bis ((1-methyl-9-propyl-9*H*-pyrido [3,4-*b*]indol-7-yl)oxy)hexane (**6c**)

White solid; yield 53%; ^1^H NMR (500 MHz, Chloroform-*d*) *δ* 8.26 (d, *J* = 5.2 Hz, 2H), 7.94 (d, *J* = 8.5 Hz, 2H), 7.71 (d, *J* = 5.2 Hz, 2H), 6.92–6.83 (m, 4H), 4.38 (t, *J* = 7.5 Hz, 4H), 4.15 (t, *J* = 6.0 Hz, 4H), 2.99 (s, 6H), 1.96–2.00 (m, 4H), 1.78–1.83 (m, 4H), 1.58–1.62 (m, 4H), 0.99 (t, *J* = 6.8 Hz, 6H); ^13^C NMR (125 MHz, Chloroform-*d*) *δ* 160.4, 143.3, 140.1, 138.2, 135.0, 129.5, 122.6, 115.3, 112.1, 109.4, 94.5, 68.2, 59.3, 29.8, 25.7, 24.2, 23.1, and 11.5; ESI–MS m/z Calcd for C_36_H_42_N_4_O_2_ [M + H]^+^ 563.3341, found 563.3386.

#### 4.2.16 1,6-bis ((9-allyl-1-methyl-9*H*-pyrido [3,4-*b*]indol-7-yl)oxy)hexane (**6d**)

Light yellow solid; yield 68%; ^1^H NMR (500 MHz, Chloroform-*d*) *δ* 8.27 (d, *J* = 5.1 Hz, 2H), 7.95 (d, *J* = 8.5 Hz, 2H), 7.71 (d, *J* = 5.2 Hz, 2H), 6.87 (dd, *J* = 8.6, 2.2 Hz, 2H), 6.84 (d, *J* = 2.2 Hz, 2H), 6.28–6.20 (m, 2H), 5.33 (dt, *J* = 4.0, 2.1 Hz, 4H), 5.28 (dd, *J* = 10.5, 1.7 Hz, 2H), 4.82–4.76 (m, 2H), 4.16 (t, *J* = 6.4 Hz, 4H), 3.00 (s, 6H), 1.85–1.74 (m, 4H), 1.50–1.38 (m, 4H); ^13^C NMR (125 MHz, Chloroform-*d*) *δ* 160.5, 142.8, 140.0, 137.1, 134.9, 133.2, 129.7, 122.3, 114.8, 113.0, 112.3, 109.4, 93.6, 68.2, 29.1, 25.8, and 22.1; ESI–MS m/z Calcd for C_36_H_38_N_4_O_2_ [M + H]^+^ 559.3028, found 559.3043.

#### 4.2.17 1,7-bis ((1,9-dimethyl-9*H*-pyrido [3,4-*b*]indol-7-yl)oxy)heptane (**7a**)

White solid; yield 60%; ^1^H NMR (500 MHz, Chloroform-*d*) *δ* 8.27 (d, *J* = 5.2 Hz, 2H), 7.96 (d, *J* = 8.6 Hz, 2H), 7.72 (d, *J* = 5.2 Hz, 2H), 6.87 (dd, *J* = 8.6, 2.2 Hz, 2H), 6.83 (d, *J* = 2.1 Hz, 2H), 4.16 (t, *J* = 6.5 Hz, 4H), 3.92 (s, 6H), 3.00 (s, 6H), 1.82–1.76 (m, 4H), 1.42–1.35 (m, 4H), 1.33 (d, *J* = 3.3 Hz, 2H); ^13^C NMR (125 MHz, Chloroform-*d*) *δ* 160.2, 143.0, 140.6, 138.2, 135.3, 129.3, 122.3, 115.2, 112.2, 108.8, 94.1, 68.7, 32.7, 29.2, 23.4, 22.9, and 20.2; ESI–MS m/z Calcd for C_33_H_36_N_4_O_2_ [M + H]^+^ 521.2872, found 521.2904.

#### 4.2.18 1,7-bis ((9-ethyl-1-methyl-9*H*-pyrido [3,4-*b*]indol-7-yl)oxy)heptane (**7b**)

White solid; yield 49%; mp 187°C–188°C; ^1^H NMR (500 MHz, Chloroform-*d*) *δ* 8.25 (d, *J* = 5.2 Hz, 2H), 7.96 (d, *J* = 8.3 Hz, 2H), 7.74 (d, *J* = 5.2 Hz, 2H), 6.92–6.86 (m, 4H), 4.51 (q, *J* = 7.0 Hz, 4H), 4.15 (t, *J* = 6.3 Hz, 4H), 2.99 (s, 6H), 1.84–1.78 (m, 4H), 1.43 (t, *J* = 6.9 Hz, 6H), 1.32–1.26 (m, 4H), ^13^C NMR (125 MHz, Chloroform-*d*) *δ* 160.4, 143.1, 140.7, 138.4, 135.5, 129.7, 122.4, 115.4, 112.3, 109.2, 93.8, 68.8, 40.3, 29.6, 23.2, 22.6, and 15.3; ESI–MS m/z Calcd for C_35_H_40_N_4_O_2_ [M + H]^+^ 549.3185, found 549.3208.

#### 4.2.19 1,7-bis ((1-methyl-9-propyl-9*H*-pyrido [3,4-*b*]indol-7-yl)oxy)heptane (**7c**)

White solid; yield 58%; mp 193°C–195°C; ^1^H NMR (500 MHz, Chloroform-*d*) *δ* 8.23 (d, *J* = 5.1 Hz, 2H), 7.92 (d, *J* = 8.5 Hz, 2H), 7.69 (d, *J* = 5.1 Hz, 2H), 6.90–6.81 (m, 4H), 4.45 (4H, q, *J* = 7.2 Hz, 4H), 4.16 (t, *J* = 6.0 Hz, 4H), 2.98 (s, 6H), 1.86–1.80 (m, 4H), 1.72–1.64 (m, 4H), 1.43–1.35 (m, 4H), 1.30–1.24 (m, 2H), 0.96 (6H, t, *J* = 7.2 Hz); ^13^C NMR (125 MHz, Chloroform-*d*) *δ* 160.5, 142.9, 140.1, 138.2, 134.9, 128.7, 122.4, 116.9, 112.4, 109.4, 93.6, 68.9, 58.6, 30.2, 25.2, 24.1, 23.5, 23.0, and 12.0; ESI–MS m/z Calcd for C_37_H_44_N_4_O_2_ [M + H]^+^ 577.3498, found 577.3533.

#### 4.2.20 1,7-bis ((9-allyl-1-methyl-9*H*-pyrido [3,4-*b*]indol-7-yl)oxy)heptane (**7d**)

Light yellow solid; yield 54%; ^1^H NMR (500 MHz, Chloroform-*d*) *δ* 8.26 (d, *J* = 5.3 Hz, 2H), 7.96 (d, *J* = 8.4 Hz, 2H), 7.72 (d, *J* = 5.2 Hz, 2H), 6.87 (dd, *J* = 8.6, 2.2 Hz, 1H), 6.84 (d, *J* = 2.2 Hz, 1H), 6.33–6.25 (m, 2H), 5.34–5.26 (m, 6H), 4.78 (dd, *J* = 17.2, 2.0 Hz, 2H), 4.18 (t, *J* = 6.0 Hz, 4H), 3.01 (s, 6H), 2.01–1.95 (m, 4H), 1.70–1.64 (m, 4H), 1.46–1.1.38 (m, 2H); ^13^C NMR (125 MHz, Chloroform-*d*) *δ* 160.6, 142.8, 140.2, 138.3, 135.2, 133.4, 129.1, 122.4, 115.2, 114.1, 112.1, 108.9, 93.4, 66.9, 47.1, 32.0, 28.9, 23.2, and 20.2; ESI–MS m/z Calcd for C_37_H_40_N_4_O_2_ [M + H]^+^ 573.3185, found 573.3214.

#### 4.2.21 1,8-bis ((1,9-dimethyl-9*H*-pyrido [3,4-*b*]indol-7-yl)oxy)octane (**8a**)

White solid; yield 55%; ^1^H NMR (500 MHz, Chloroform-*d*) *δ* 8.26 (d, *J* = 5.2 Hz, 2H), 7.98 (d, *J* = 8.6 Hz, 2H), 7.72 (d, *J* = 5.2 Hz, 2H), 6.88 (dd, *J* = 8.6, 2.2 Hz, 2H), 6.84 (d, *J* = 2.1 Hz, 2H), 4.14 (d, *J* = 6.3 Hz, 4H), 4.00 (s, 6H), 3.01 (s, 6H), 1.92–1.84 (m, 4H), 1.60–1.56 (d, *J* = 6.8 Hz, 4H), 1.52–1.49 (s, 4H); ^13^C NMR (125 MHz, Chloroform-*d*) *δ* 160.2, 143.1, 140.2, 137.8, 134.8, 129.5, 122.5, 114.9, 112.2, 109.3, 93.4, 68.0, 39.5, 29.4, 29.1, 25.2, and 21.3; ESI–MS m/z Calcd for C_34_H_38_N_4_O_2_ [M + H]^+^ 535.3028, found 535.3059.

#### 4.2.22 1,8-bis ((9-ethyl-1-methyl-9*H*-pyrido [3,4-*b*]indol-7-yl)oxy)octane (**8b**)

White solid; yield 61%; ^1^H NMR (500 MHz, Chloroform-*d*) *δ* 8.28 (d, *J* = 5.2 Hz, 2H), 7.96 (d, *J* = 8.5 Hz, 2H), 7.73 (d, *J* = 5.2 Hz, 2H), 6.88 (dd, *J* = 8.5, 2.1 Hz, 2H), 6.86 (d, *J* = 2.0 Hz, 2H), 4.43 (q, *J* = 7.4 Hz, 4H), 4.16 (t, *J* = 6.3 Hz, 4H), 3.01 (s, 6H), 2.02–1.95 (m, 4H), 1.89–1.69 (m, 6H), 1.45 (t, *J* = 7.4 Hz, 4H), 1.34–1.26 (m, 4H); ^13^C NMR (125 MHz, Chloroform-*d*) *δ* 160.2, 143.0, 140.6, 138.2, 135.3, 129.3, 122.3, 115.2, 112.2, 108.8, 94.1, 68.2, 44.6, 32.7, 29.2, 23.4, 20.2, and 13.90. ESI–MS m/z Calcd for C_36_H_42_N_4_O_2_ [M + H]^+^ 563.3341, found 563.3348.

#### 4.2.23 1,8-bis ((1-methyl-9-propyl-9*H*-pyrido [3,4-*b*]indol-7-yl)oxy)octane (**8c**)

White solid; yield 52%; ^1^H NMR (500 MHz, Chloroform-*d*) *δ* 8.27 (d, *J* = 5.1 Hz, 2H), 7.95 (d, *J* = 8.5 Hz, 2H), 7.72 (d, *J* = 5.1 Hz, 2H), 6.90–6.84 (m, 4H), 4.46 (q, *J* = 7.1 Hz, 4H), 4.15 (t, *J* = 6.2 Hz, 4H), 3.00 (s, 6H), 1.99–1.93 (m, 4H), 1.86–1.82 (m, 4H), 1.68–1.60 (m, 4H), 1.36–1.28 (m, 4H), 0.99 (t, *J* = 6.9 Hz, 6H); ^13^C NMR (125 MHz, Chloroform-*d*) *δ* 160.5, 142.9, 140.5, 138.1, 135.0, 129.1, 122.3, 115.4, 112.0, 109.0, 93.6, 67.9, 54.6, 32.4, 26.8, 24.4, 22.2, 23.4, and 12.0; ESI–MS m/z Calcd for C_38_H_46_N_4_O_2_ [M + H]^+^ 591.3654, found 591.3678.

#### 4.2.24 1,8-bis ((9-allyl-1-methyl-9*H*-pyrido [3,4-*b*]indol-7-yl)oxy)octane (**8d**)

Light yellow solid; yield 54%; ^1^H NMR (500 MHz, Chloroform-*d*) *δ* 8.26 (d, *J* = 5.2 Hz, 2H), 7.96 (d, *J* = 8.4 Hz, 2H), 7.72 (d, *J* = 5.2 Hz, 2H), 6.87 (dd, *J* = 8.6, 2.2 Hz, 2H), 6.84 (d, *J* = 2.2 Hz, 2H), 6.30–6.22 (m, 2H), 5.32 (dt, *J* = 4.0, 2.1 Hz, 4H), 5.30 (dd, *J* = 10.8, 2.0 Hz, 2H), 4.80 (dd, *J* = 17.1, 2.0 Hz, 2H), 4.16 (4H, t, *J* = 6.0 Hz, 4H), 2.99 (s, 6H), 2.02–1.97 (m, 4H), 1.79–1.74 (m, 4H), 1.59–1.51 (m, 4H); ^13^C NMR (125 MHz, Chloroform-*d*) *δ* 160.8, 143.0, 140.5, 138.5, 135.5, 133.6, 129.3, 122.5, 115.4, 114.3, 112.2, 109.1, 93.7, 67.5, 47.3, 32.2, 29.3, 23.4, and 20.3; ESI–MS m/z Calcd for C_38_H_42_N_4_O_2_ [M + H]^+^ 587.3341, found 587.3374.

### 4.3 Inhibition of AChE and BuChE

The Ellman’s assay was utilized to assess the inhibitory potential of novel bis(7)-harmine derivatives against *h*AChE and *h*BuChE. 50 *μ*L of *h*AChE (0.02 unit/mL) or *h*BChE (0.02 unit/mL) were incubated with 10 *μ*L of the compound in 96-well plates at 37°C for 6 min. Subsequently, 30 *μ*L of a substrate solution containing acetylthiocholine iodide (ATCI) or butyrylthiocholine iodide (BTCI) at a concentration of 0.01 M was added, and the mixture was further incubated at 37°C for an additional duration of 12 min. Finally, the activity was measured by adding 150 *μ*L of a solution containing 5,5′−dithiobis (2-nitrobenzoic acid) (DTNB) at a concentration of 0.01 M, followed by measuring absorbance at a wavelength of 415 nm using an Evolution 300 PC UV-Vis Spectrophotometer.

### 4.4 Inhibition of *h*MAO-A and *h*MAO-B

The inhibitory activity of these derivatives on both recombinant *h*MAO-A and *h*MAO-B (Sigma-Aldrich) was assessed using a fluorescence-based method as previously described (Giovannuzzi et al., 2024). Briefly, the compounds under investigation and the reference inhibitor were preincubated with kynuramine at 37°C for 10 min in 96-well microplates. Then, the reaction was started with the addition of *h*MAO-A or *h*MAO-B. Initial velocities were determined spectrophotometrically in a microplate reader at 37°C by measuring the formation of 4-hydroxyquinoline at 316 nm, over a period of at least 30 min. The enzymatic reactions were terminated by adding 400 *μ*L of 2 N NaOH and 1,000 *μ*L of water, followed by centrifugation at 16,000 g for 10 min. Subsequently, the concentrations of MAOs that produced 4-hydroxyquinoline were determined by measuring the fluorescence of the supernatant using a Varioskan Flash Multimode Reader (PerkinElmer) with excitation and emission wavelengths set at 310 nm and 400 nm, respectively. The IC_50_ values were calculated from dose-response curves and expressed as the mean ± standard deviation. These values were determined based on at least three independent experiments, each performed in triplicate.

### 4.5 Inhibition of A*β*_1−42_ self−aggregation

The thioflavin T (Th-T) fluorescence assay was used to assess the inhibition of A*β*_1−42_ self-aggregation. A*β*_1-42_ (20 *μ*M final concentration) was incubated with test compounds (20 *μ*M final concentration) in a 50 mM phosphate-buffered saline (PBS, pH 7.4) at 37°C for 24 h. Subsequently, the reaction was terminated to a final volume of 200 *μ*L using Th-T (10 *μ*M) solution. The detailed procedure followed our previous work (Du et al., 2023).

### 4.6 Inhibition of AChE induced A*β*_1−42_ aggregation assay

The inhibition of AChE-induced aggregation of the A*β*_1-42_ peptide was achieved by co-incubating synthesized compounds (at concentrations of 0.1 *μ*M and 1 *μ*M) with AChE (at a concentration of 10 *μ*M). Control experiments were conducted in the absence of test compounds. The aggregation process of the A*β*_1-42_ peptide was monitored at 37°C for a duration of 24 h using Th-T, with an excitation wavelength set at 446 nm and emission ranging from 490 nm.

### 4.7 Assessment of *in vitro* cytotoxicity

The cytotoxicity of the compounds was evaluated using an MTT assay, following a previously described protocol (Ellman et al., 1961). Briefly, SHSY5Y cells were cultured in 96-well plates at a density of 1.0 × 10^4^ cells per well for 24 h. Subsequently, the cells were treated with various concentrations of each compound (0.1, 1, 10, and 100 *μ*M) for a duration of 48 h. After incubation, the culture medium was removed and replaced with 100 *μ*L of MTT solution which was then incubated at 37°C for 1 hour. Following this incubation period, the MTT solution was substituted with 100 mL of DMSO and further incubated at room temperature for 10 minutes to dissolve formazan crystals formed by viable cells. Sorensen Buffer (5 mL) was added subsequently followed by measuring absorbance at a wavelength of 570 nm to determine cell viability based on calculated values obtained from control samples without compound treatment. This experimental procedure was repeated independently three times.

### 4.8 Protection of SHSY5Y cells against damage induced by A*β*_1−42_

The SHSY5Y cells were seeded in 96-well plates at a density of 1 × 10^4^ cells per well and incubated at 37°C for 24 h. Each compound was dissolved in DMSO, followed by direct dilution in the cell culture medium to achieve final concentrations of 1 *μ*M, 5 *μ*M, and 10 *μ*M. A*β*_1−42_ was added to each well at a concentration of 5 *μ*M. The cells were then further incubated at 37°C for an additional period of 48 h. Cell viability was subsequently assessed using the MTT assay protocol (Ellman et al., 1961).

### 4.9 ADMET study

The ADMETlab 2.0 (https://admetmesh.scbdd.com), preADMET (https://preadmet.qsarhub.com/) and ProTox−II (http://tox.charite.de/protox_II) were employed to predict ADME properties and toxicity, utilizing a dedicated webserver for this purpose (Xiong et al., 2021; Banerjee et al., 2018).

### 4.10 Molecular docking

The Surflex-Dock program in Sybyl-X 2.0 Software was employed for molecular docking, with the ligand structures being sketched using the Sybyl package. Atom types were validated, hydrogen atoms were added, and Gasteiger-Marsili charges were assigned using Sybyl-X 2.0 Software. The protein structures of *h*AChE (PDB code: 4EY7), *h*MAO-B (PDB code: 2V60) and A*β*_1-42_ (PDB code: 1IYT) were obtained from the RCSB Protein Data Bank website (https://www.rcsb.org/). To facilitate molecular docking studies, the ligand was extracted from the crystal structure, water molecules were eliminated, and side-chain amides were verified before generating a protomold for further analysis. Visualization of docking results was aided by PyMOL and LigPus software tools.

## Data Availability

The original contributions presented in the study are publicly available. This data can be found here: doi: 10.6084/m9.figshare.28254113.
