# Supplementary material for: Bis(7)-harmine derivatives as potential multi-target anti-Alzheimer agents
Source: Front Chem. 2025 Jan 29;13:1545908. doi: 10.3389/fchem.2025.1545908 (PMC11813896; doi:10.3389/fchem.2025.1545908)
Supplement: Supplementary file 1 [file DataSheet1.doc]

**Supporting Information**

**B****is(7)-harmine derivatives as potential multi-target anti-Alzheimer agents**

Hongtao Du ^a, b, c^*, Fang Ma ^a, b^, Yuanyuan Cao ^a^, Miaoyan Bai ^a^, Yang Xu ^a^, Xinyi Gao ^a^, Ziyi Yang ^a^, Yan Yan ^a, d^

^a^ Shaanxi Key Laboratory of Chinese Jujube, College of Life Sciences, Yan’an University, Yan’an 716000, Shaanxi, China;

^b^ Shaanxi Qi Yuan Kang Bo Biotechnology Co. LTD, Tongchuan 727000, Shaanxi, China;

^c^ College of Life Science, Xinyang Normal University, Xinyang 464000, China;

^d^ Northwest A&F University, Yangling 712100, Shaanxi Province, China.

[duhongtao8410@163.com](mailto:duhongtao8410@163.com) (Hongtao Du)

[mafang_1984@126.com](mailto:mafang_1984@126.com) (Fang Ma)

[15389199603@163.com](mailto:15389199603@163.com) (Yuanyuan Cao)

[bmy18049627644@163.com](mailto:bmy18049627644@163.com) (Miaoyan Bai)

[yangxuyanda@126.com](mailto:yangxuyanda@126.com) (Yang Xu)

[xinyigao0810@163.com](mailto:xinyigao0810@163.com) (Xinyi Gao)

[ZyYang4219@126.com](mailto:ZyYang4219@126.com) (Ziyi Yang)

[yanyan081124@163.com](mailto:yanyan081124@163.com) (Yan Yan)

*** Corresponding author:** [duhongtao8410@163.com](mailto:duhongtao8410@163.com) (H.T. Du)

**^1^H NMR and ^13^C NMR spectra of representative compounds**

**
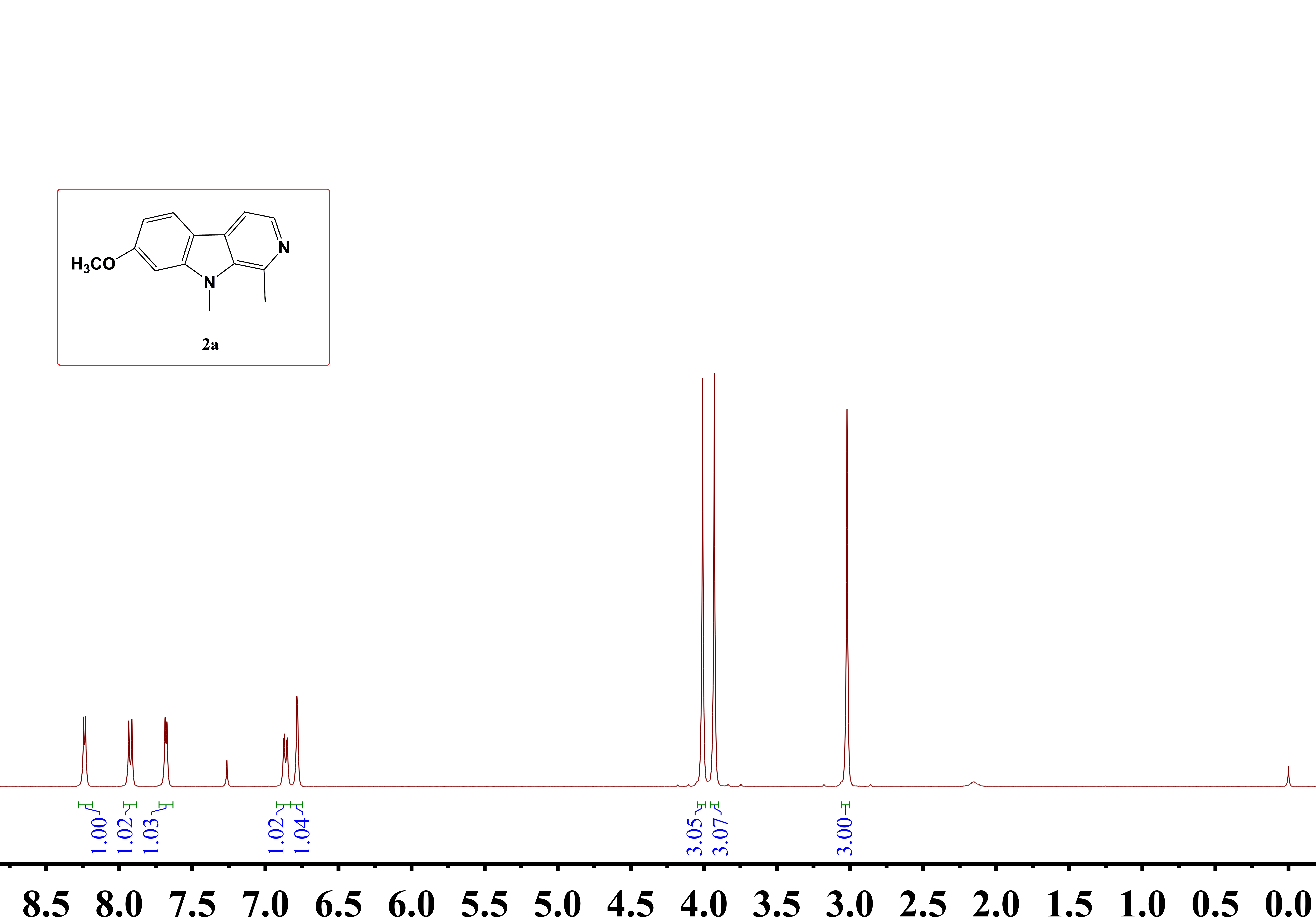
**

**
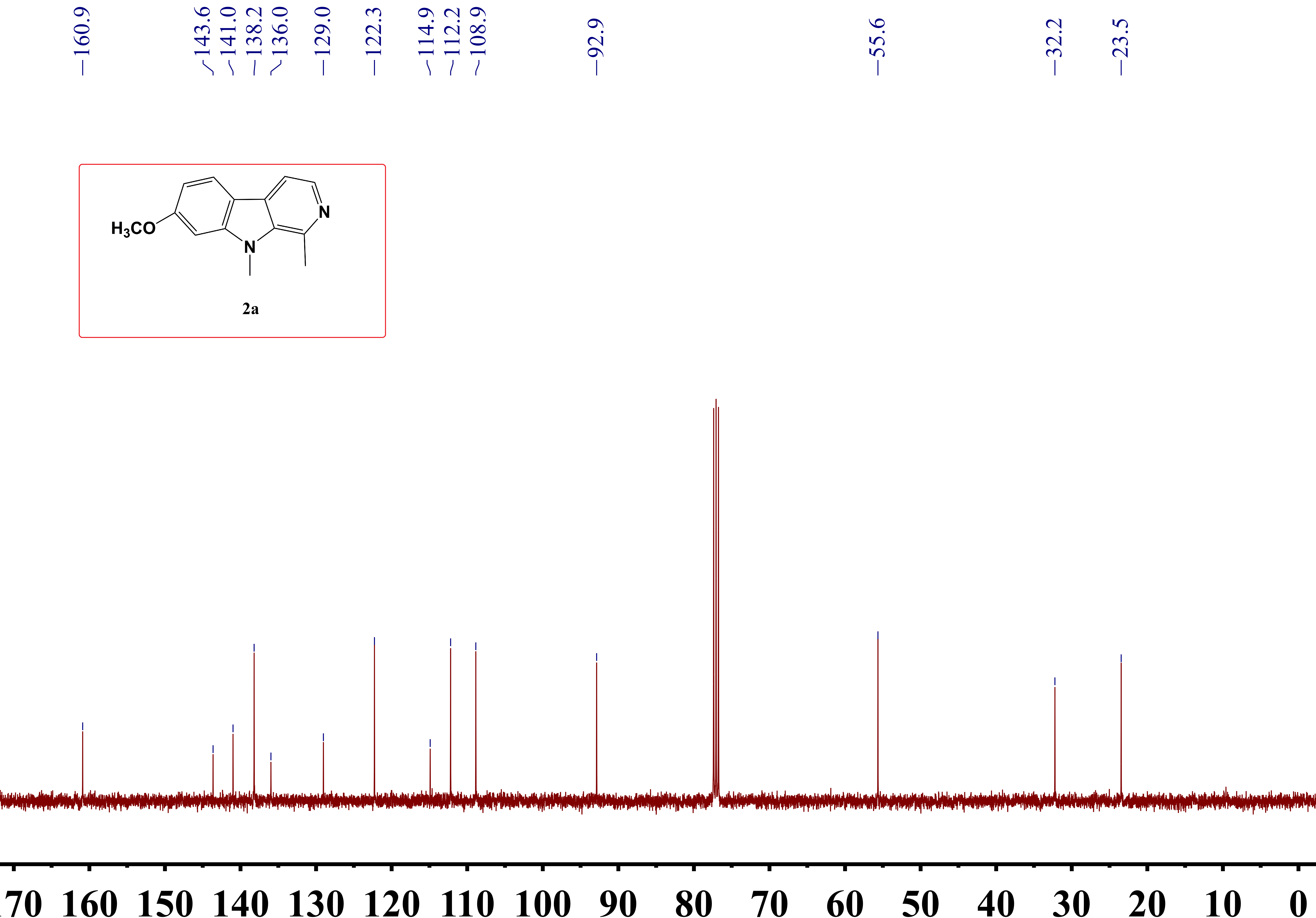
**

**2a**

**
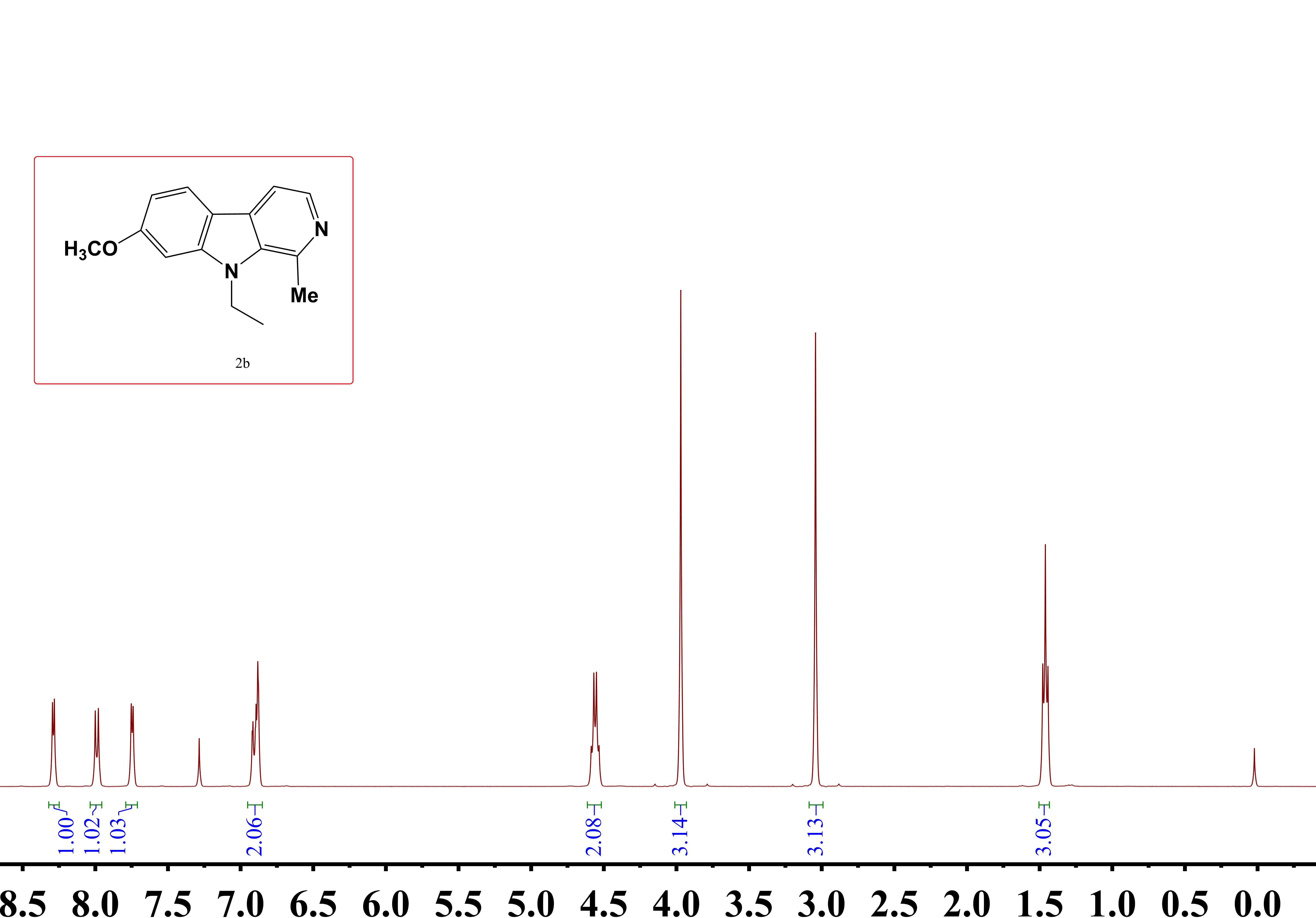
**

**
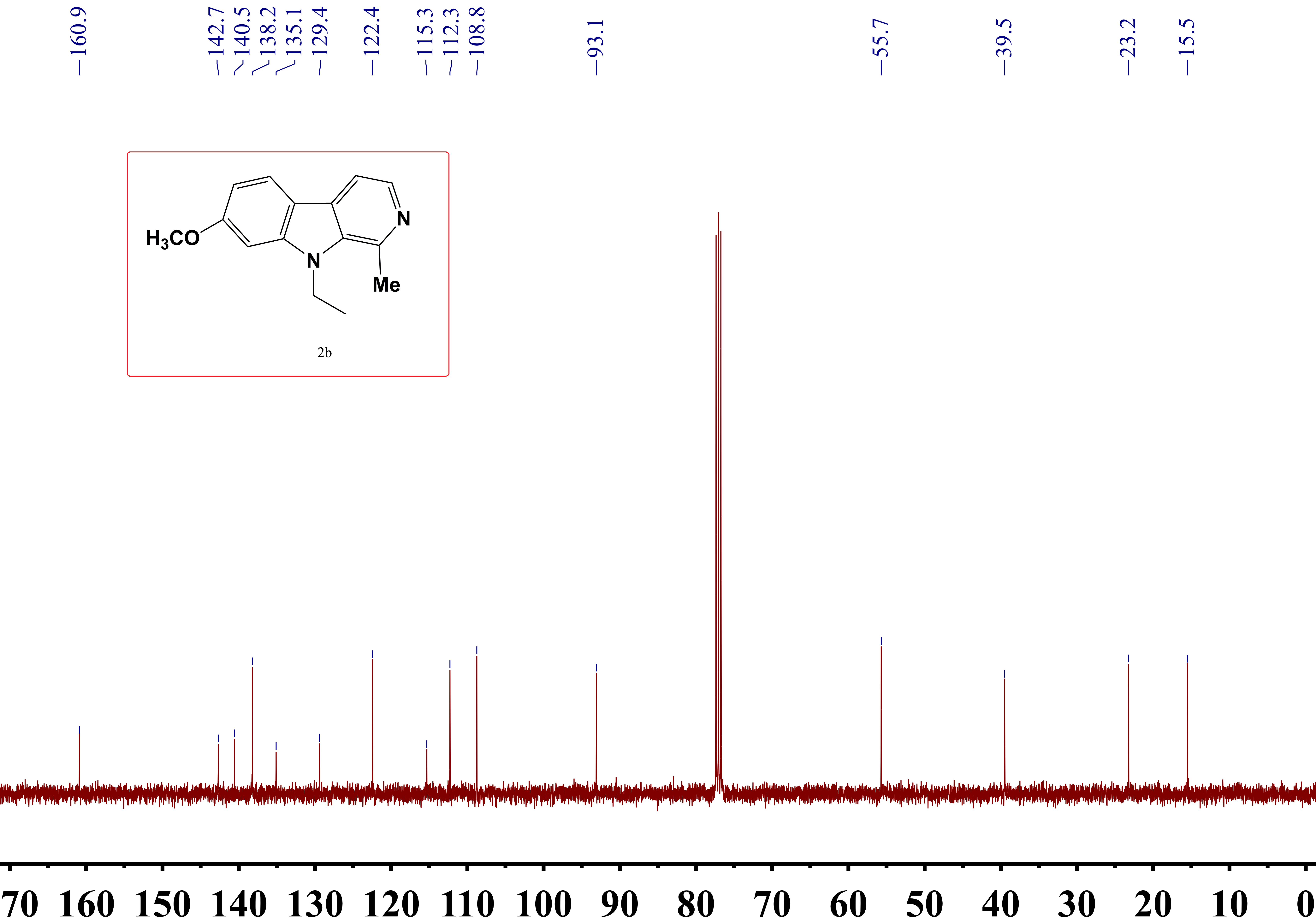
**

**2b**

**
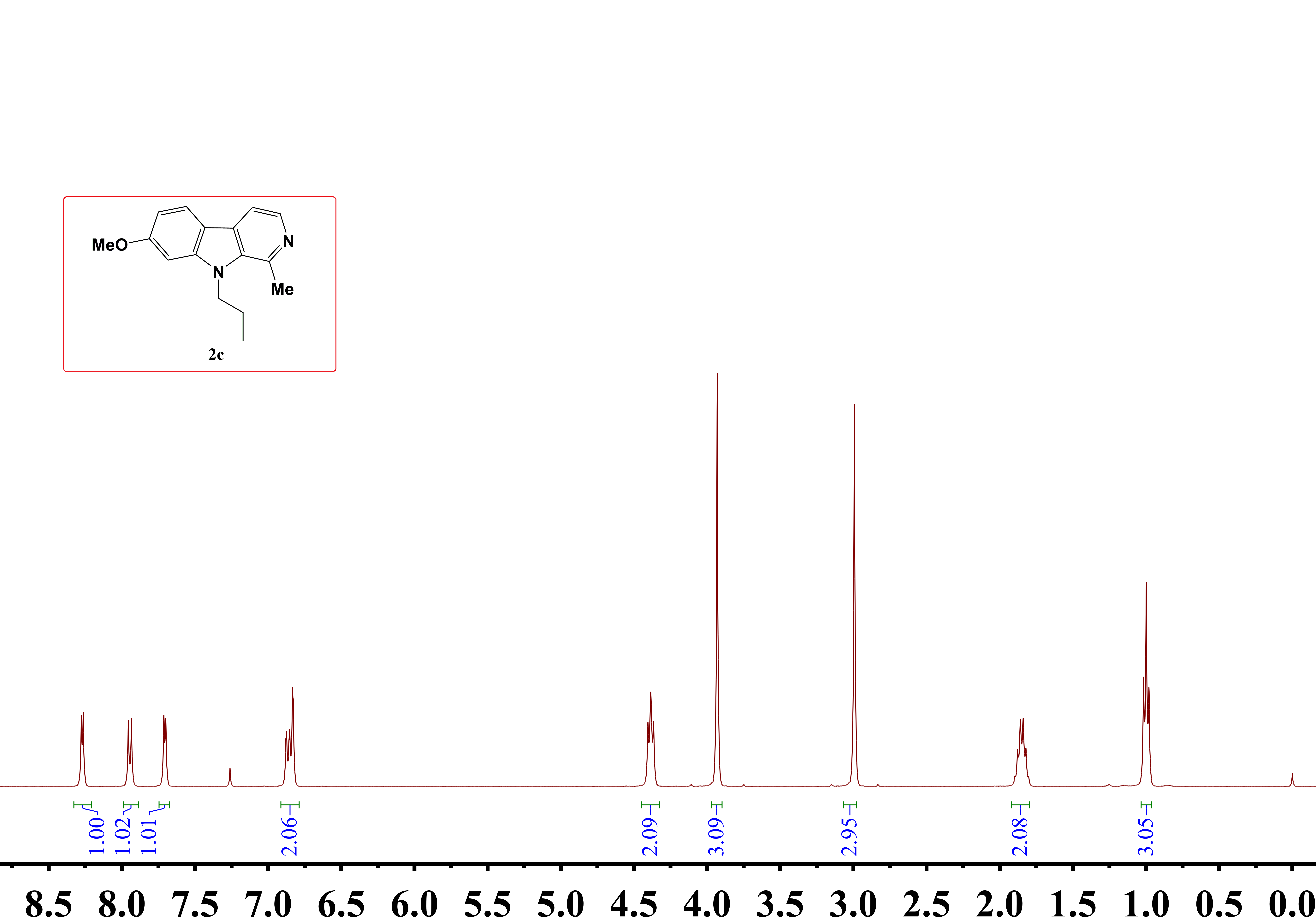
**

**
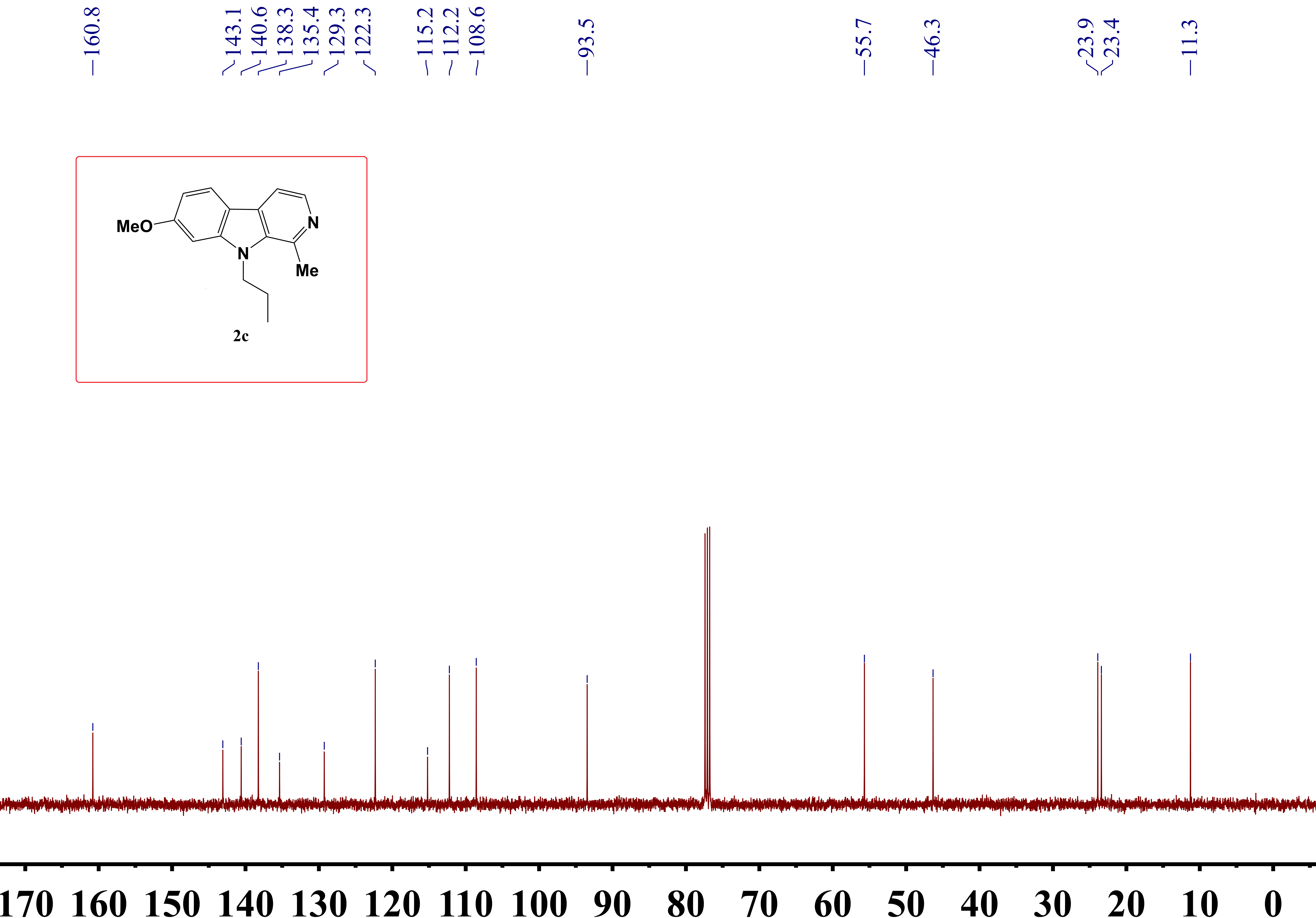
**

**2c**

**
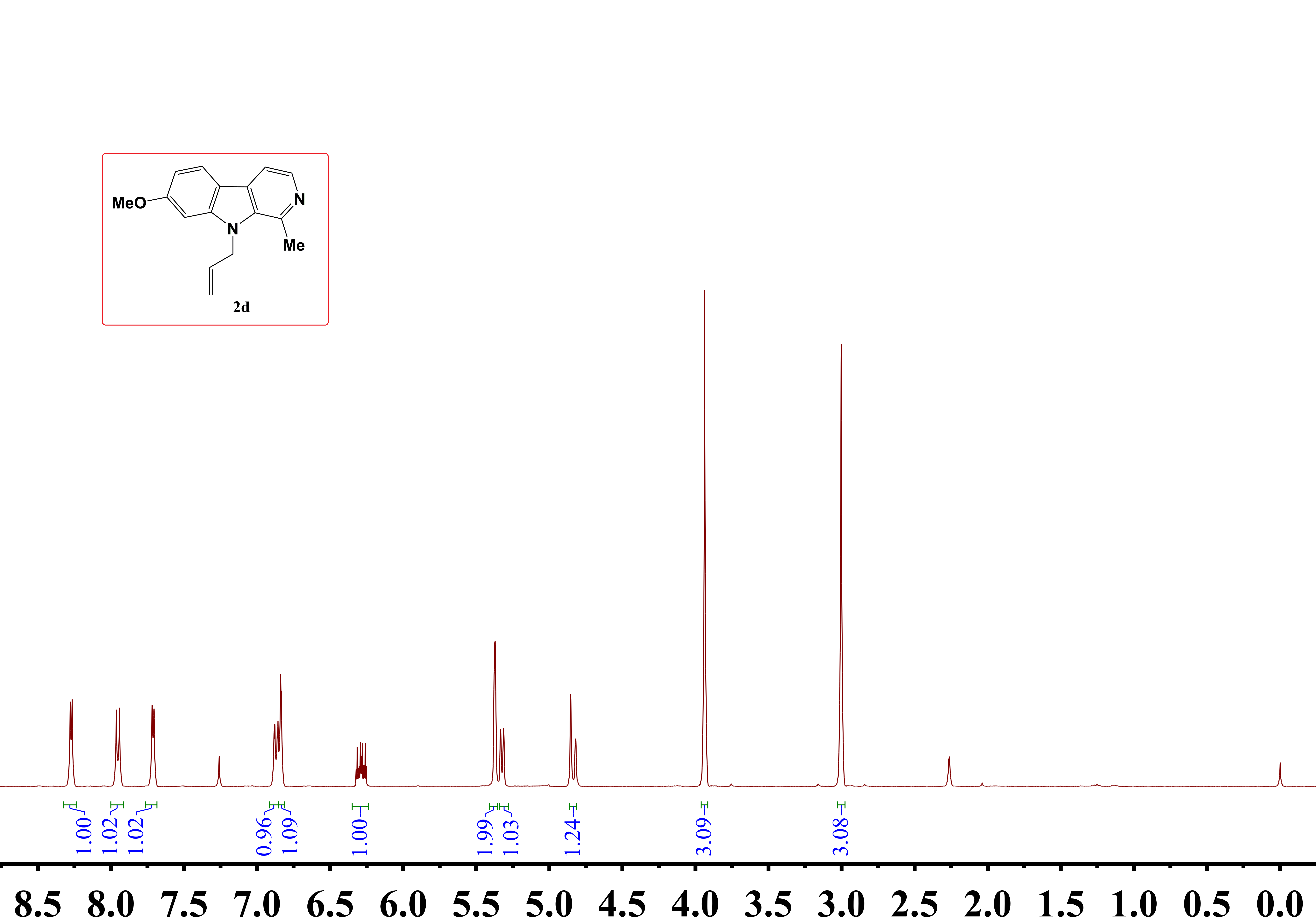
**

**
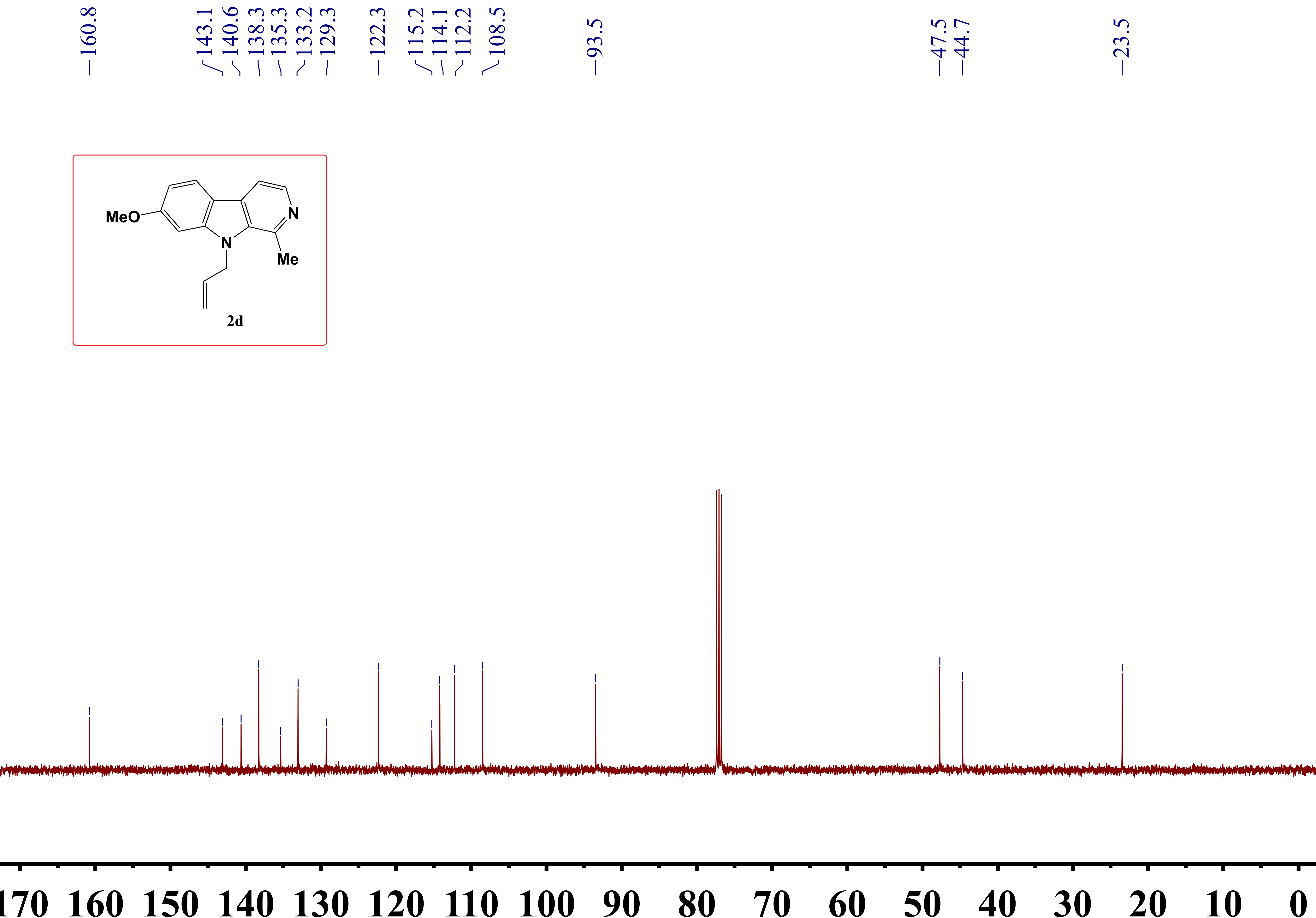
**

**2d**

**
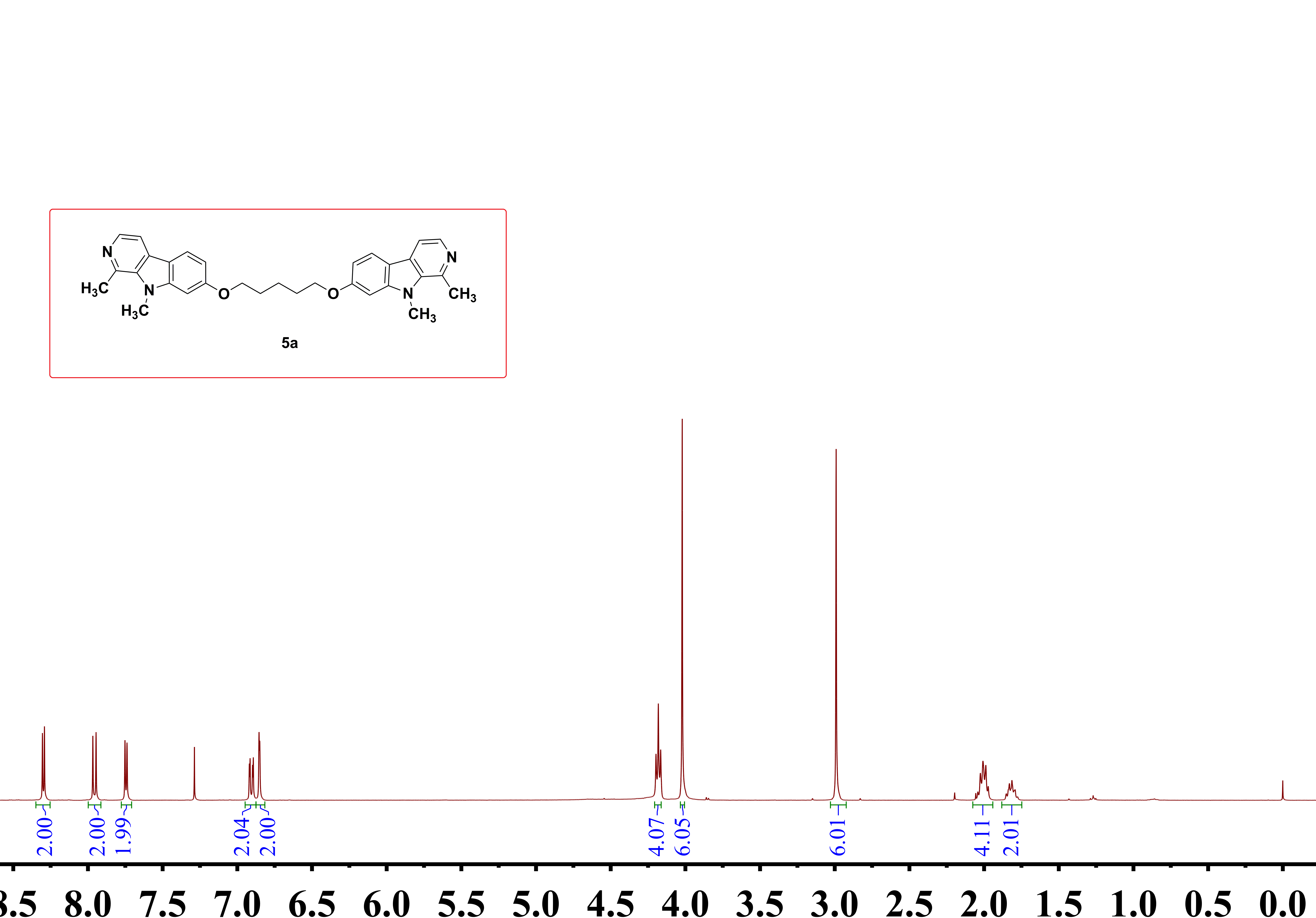
**

**
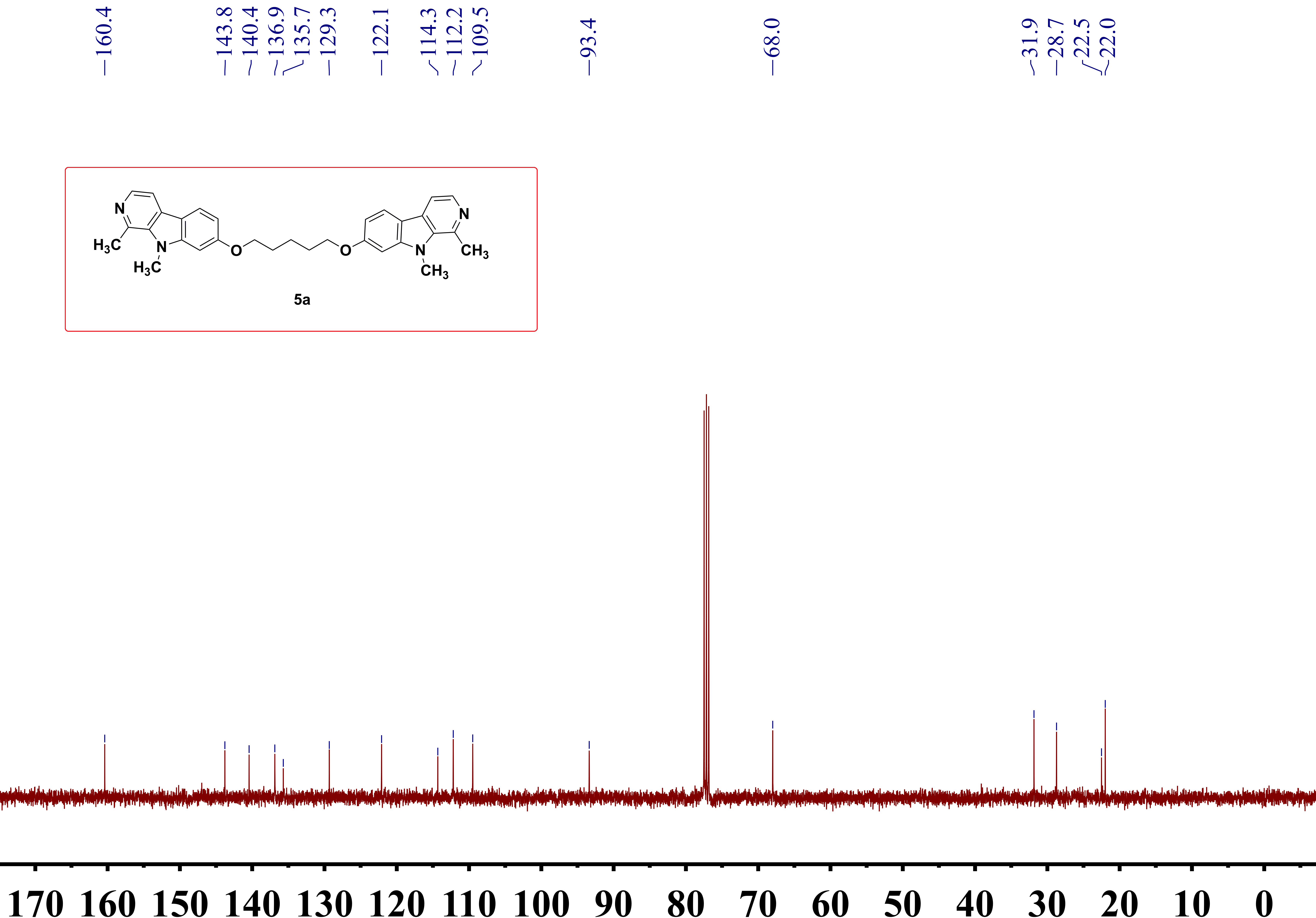
**

**5a**

**
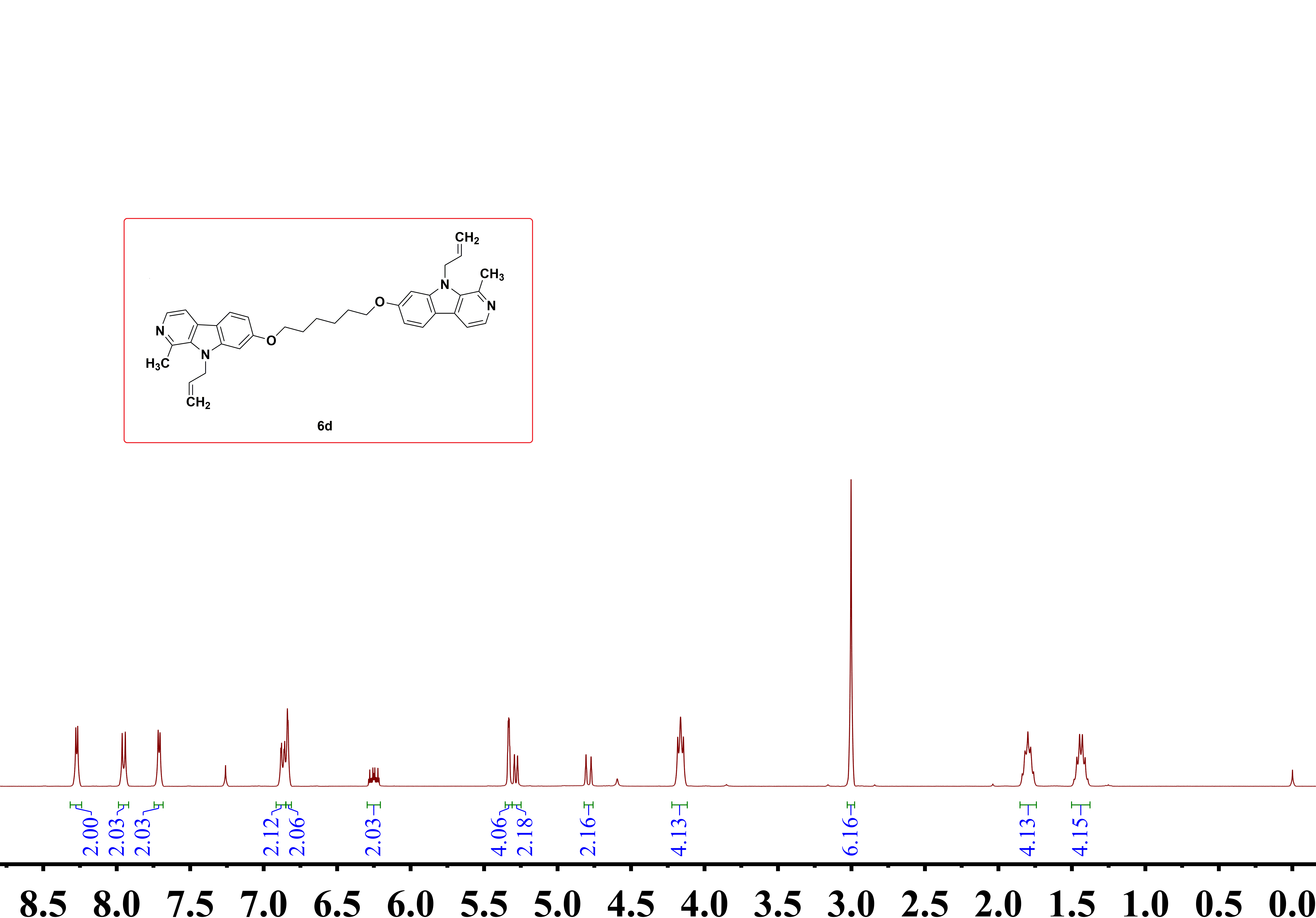
**

**
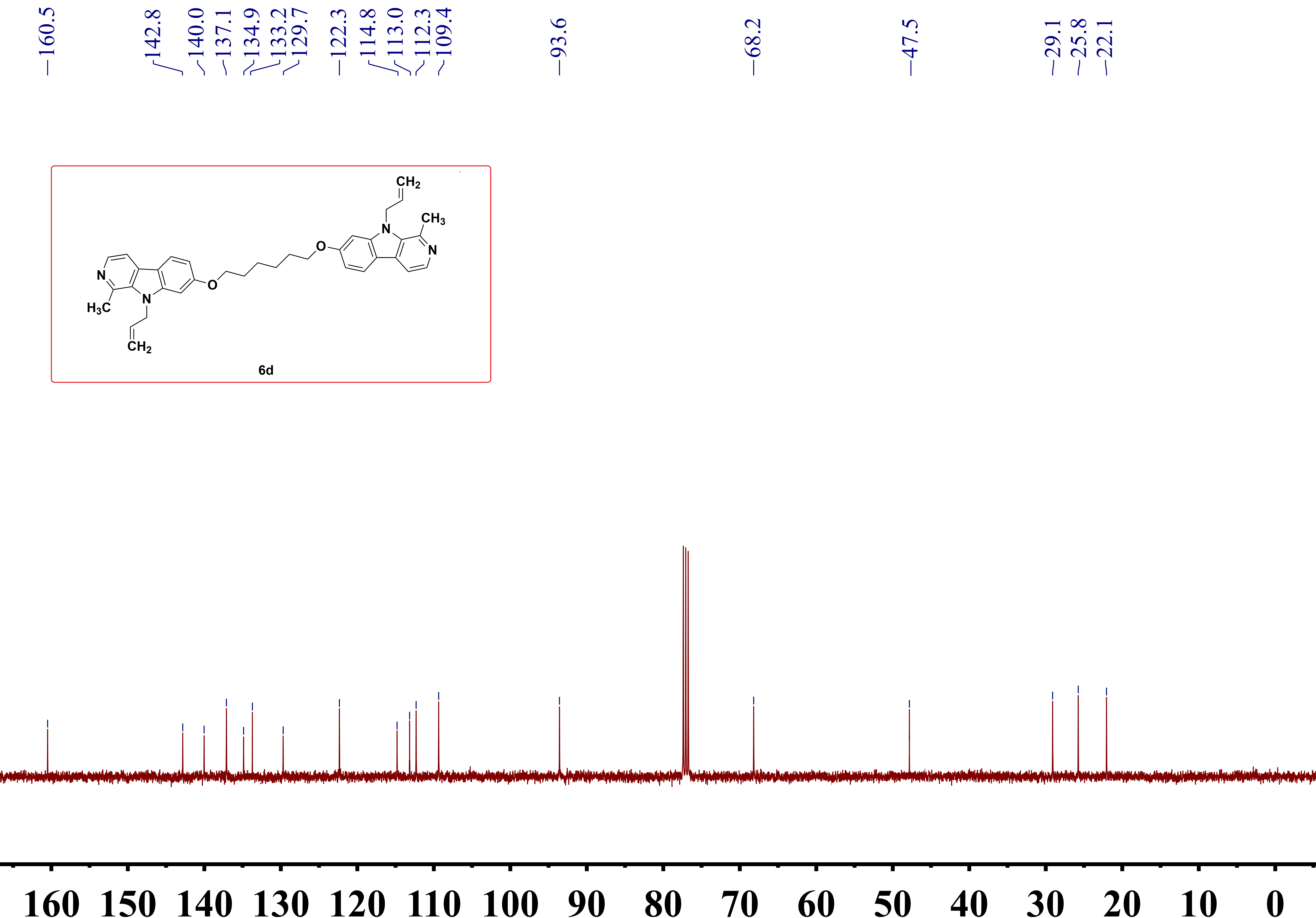
**

**6d**

**
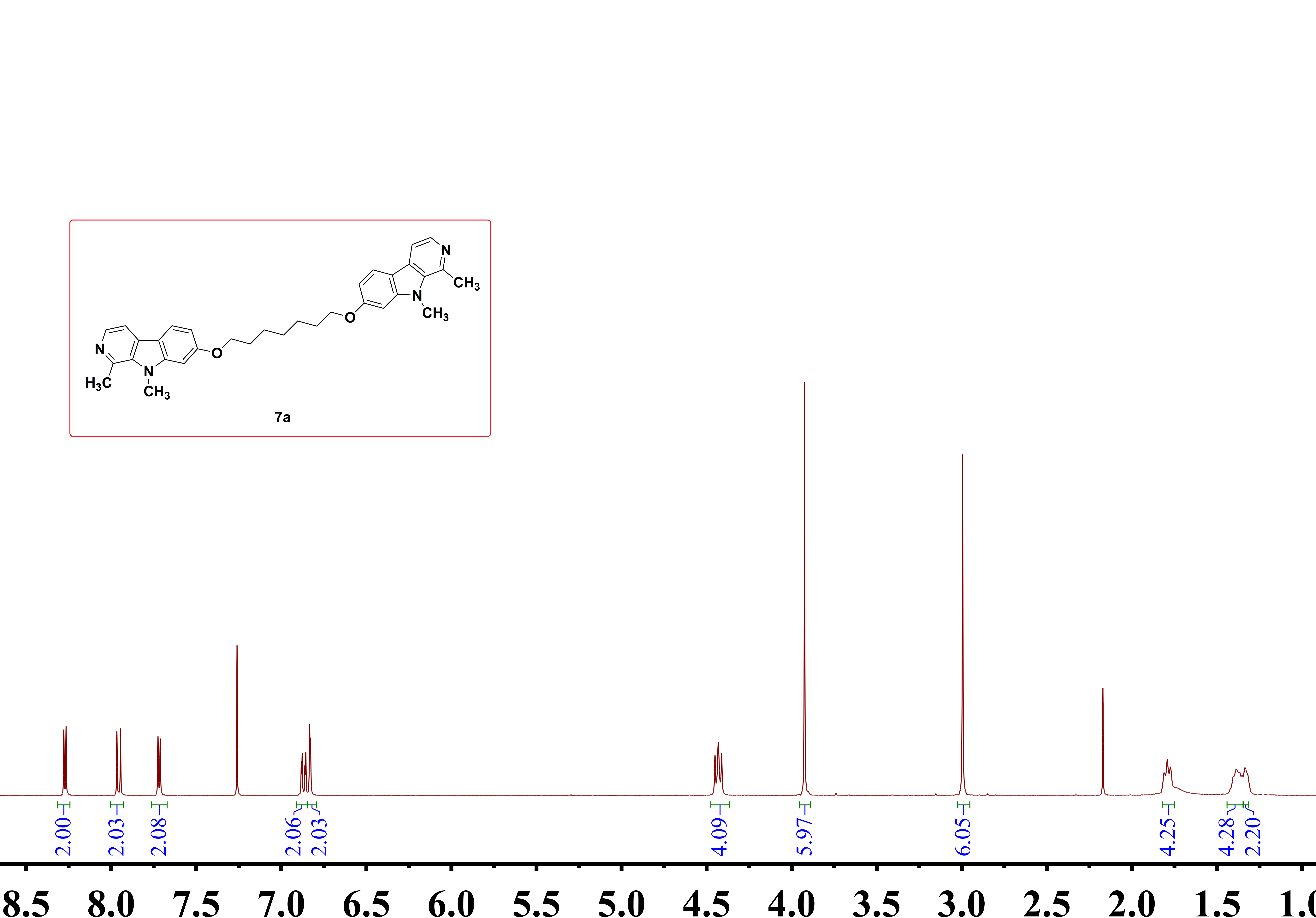
**

**
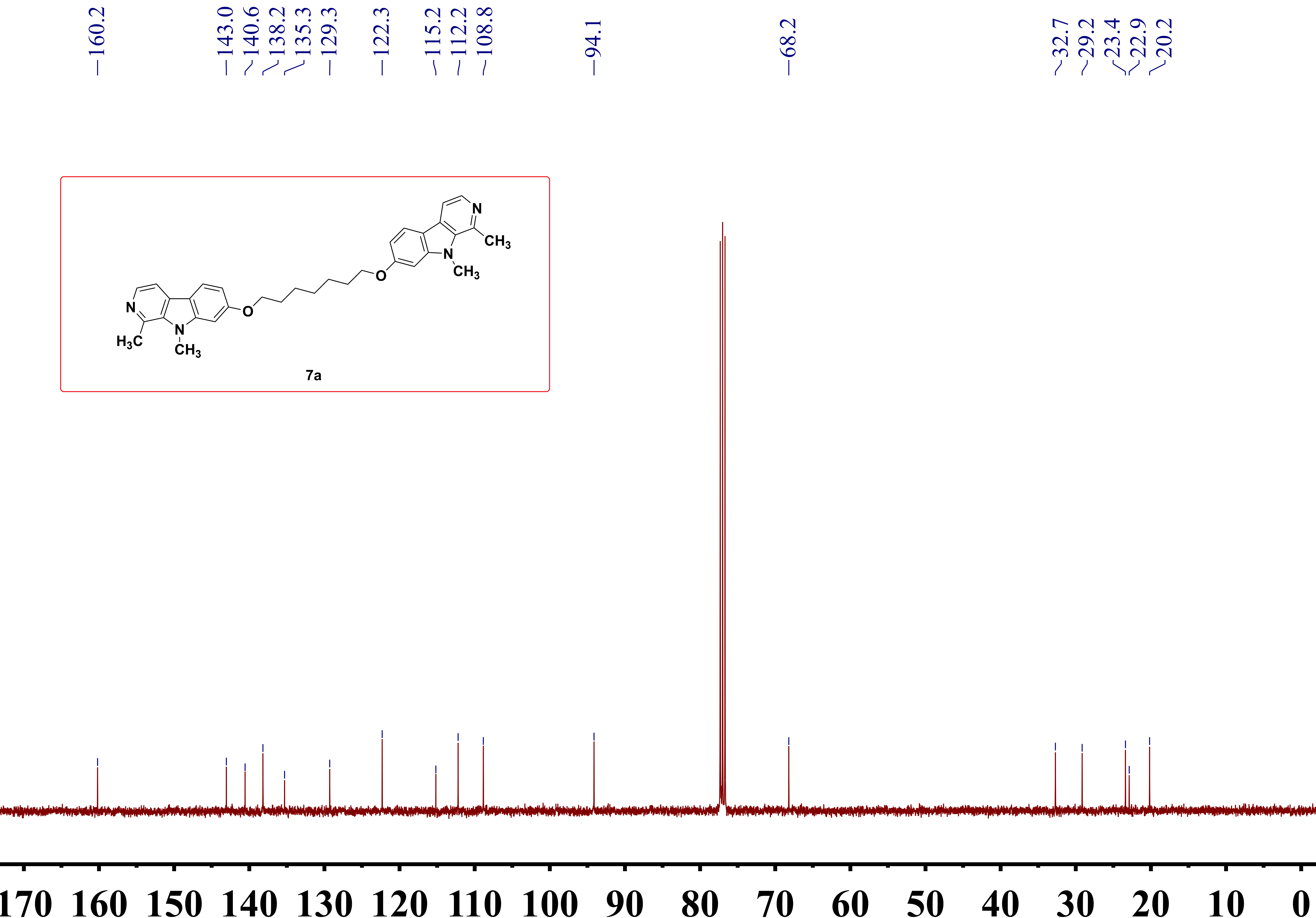
**

**7a**

**
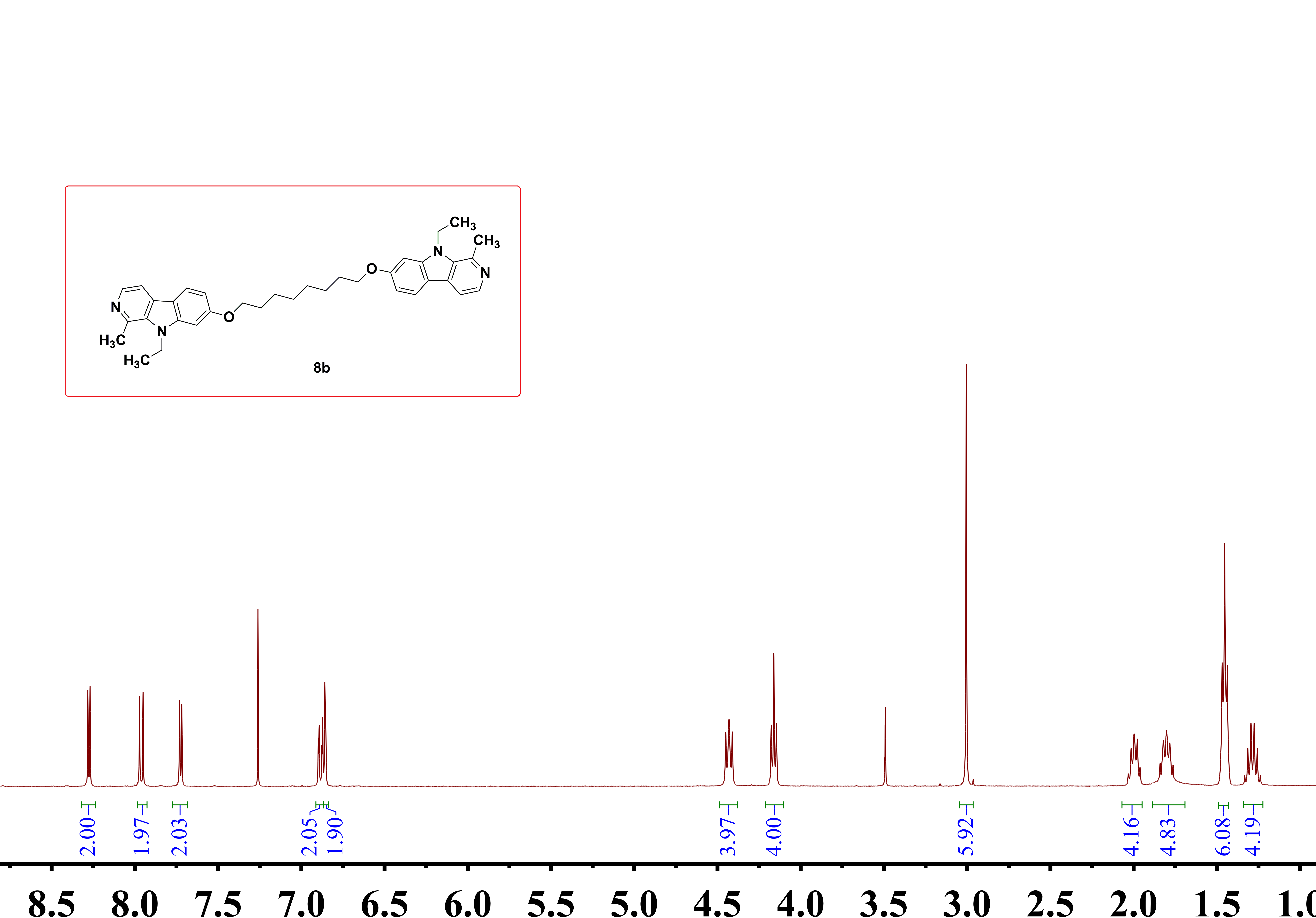
**

**
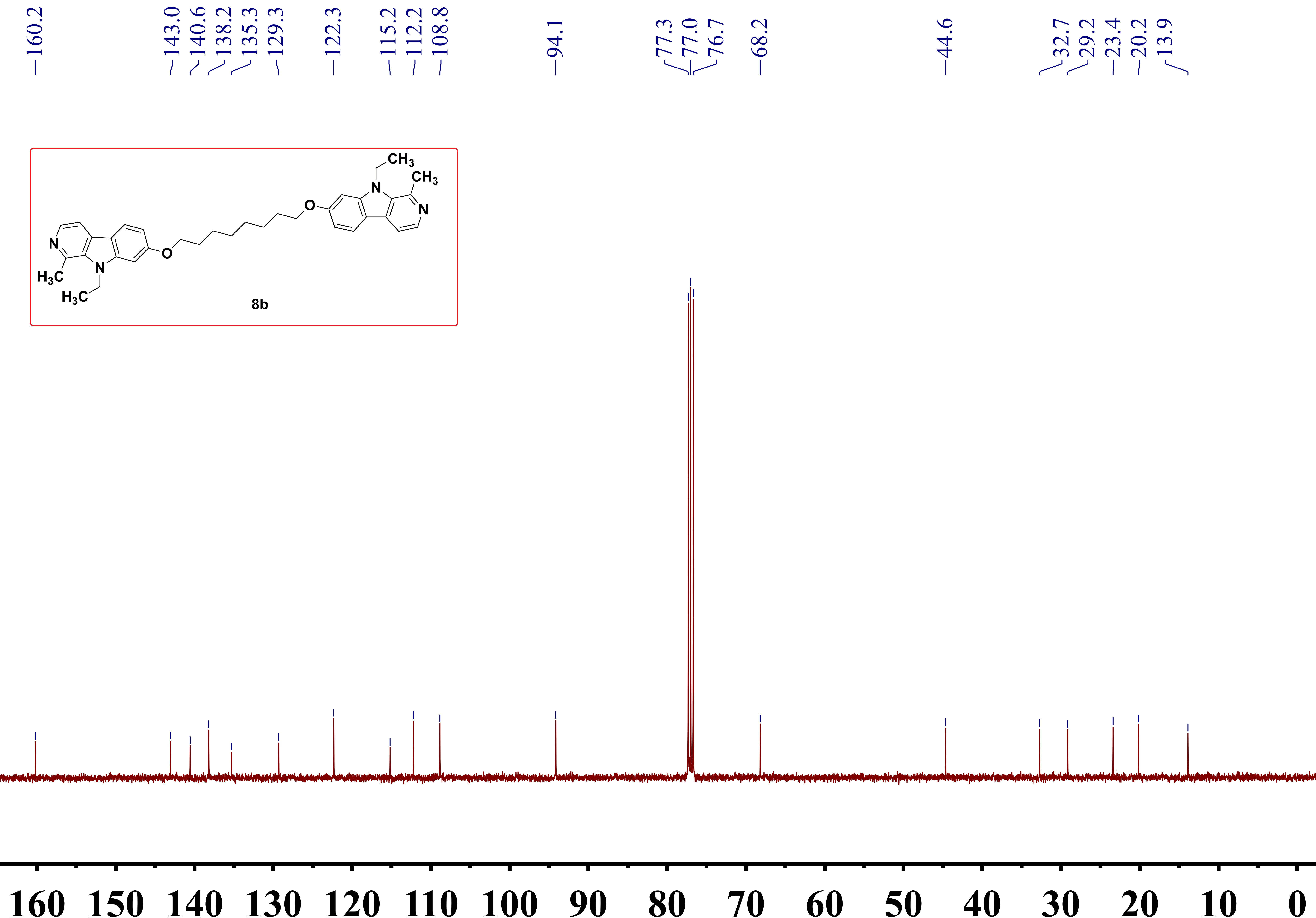
**

**8b**
